# Effectiveness and sustainability of neonatal resuscitation training programs in reducing neonatal mortality rates in low- and middle-income countries: a systematic review

**DOI:** 10.1080/16549716.2026.2757387

**Published:** 2026-11-05

**Authors:** Kurnia Rachmawati, Victoria J. Kain, Elizabeth M. Forster

**Affiliations:** a School of Nursing and Midwifery, Griffith University, Brisbane, Queensland, Australia; b School of Nursing, Universitas Lambung Mangkurat, Banjarbaru, Indonesia; c School of Nursing and Midwifery, University of Southern Queensland, Ipswich, Queensland, Australia

**Keywords:** Birth asphyxia, Helping Babies Breathe, newborn care, training effectiveness, SDG 3: Good health and well-being

## Abstract

Globally, 75% of neonatal deaths occur in the first week of life. Despite a 52% reduction in the neonatal mortality rate (1990–2022), significant disparities persist in low- and middle-income countries (LMICs). This systematic review synthesises evidence on the effectiveness and sustainability of neonatal resuscitation training programs in LMICs, with a focus on training programs, design, provider competency outcomes, and sustainability features. Systematic searches of five electronic databases (Embase, PubMed, MEDLINE (Ovid), Scopus, and CINAHL) were conducted in accordance with PRISMA guidelines to identify studies published between January 2020 and January 2025. Studies evaluating neonatal resuscitation training for healthcare providers in LMICs and reporting competency or mortality outcomes were eligible. Risk of bias and methodological quality were assessed using the Mixed Methods Appraisal Tool. Fifty-two studies across 23 LMICs met the inclusion criteria. The majority demonstrated immediate improvements in provider knowledge and skills, although the magnitude and durability of effects varied across study designs and settings. Helping Babies Breathe was the most evaluated training program. Multi-component interventions incorporating training, mentorship, and quality improvement were associated with greater mortality reductions than training alone in narrative comparisons. Limited long-term follow-up constrains sustainability conclusions. Neonatal resuscitation training programs improve healthcare providers’ competencies and, when implemented with system-level support, are associated with reduced mortality. Future research should incorporate validated sustainability assessment frameworks and extended follow-up to strengthen the evidence base for long-term program impact. This review was registered on PROSPERO (CRD42024623116). This review received no external funding.

## Paper Context

- **Main findings**: Neonatal resuscitation training programs in low- and middle-income countries consistently improve healthcare providers’ competencies; multi-component interventions integrating training, mentorship, and quality improvement achieve better outcomes than standalone training.
- **Added knowledge**: This review synthesises post-2020 evidence from 52 studies across 23 low- and middle-income countries, capturing pandemic-era adaptations and identifying that no included studies employed validated sustainability assessment tools, representing a critical gap requiring urgent attention.
- **Global health impact for policy and action**: Policymakers should prioritise health system-integrated, multi-component neonatal resuscitation training programs over standalone approaches, and future programs must incorporate validated sustainability assessment frameworks and extended follow-up to advance progress toward ending preventable neonatal mortality by 2030.

## Background

The neonatal period, defined as the 28-day period following birth [1,2], represents a critical window of vulnerability for infants [1,3]. According to the World Health Organisation, an estimated 2.3 million newborn deaths occurred globally in 2022 [4]. Sub-Saharan Africa and South Asia record the highest neonatal mortality rates globally, with 27 and 22 deaths per 1,000 live births, respectively [4]. This contrasts sharply with high-income countries, such as Australia and New Zealand, where neonatal mortality is very low at 2.3 deaths per 1000 live births [5]. The global neonatal mortality rate has declined by 52% between 1990 and 2022, from 37 to 17 deaths per 1,000 live births [6]. Despite global initiatives to reduce neonatal mortality, the three primary causes persist: preterm birth, intrapartum complications leading to birth asphyxia, and neonatal sepsis [7]. Although these causes are difficult to eliminate completely, their impact can be substantially reduced through targeted interventions, particularly effective resuscitation at birth [8].

The implementation of neonatal resuscitation is a key strategy for reducing neonatal mortality and supporting the Sustainable Development Goal targets related to newborn survival [9–12]. Birth asphyxia is a leading cause of neonatal mortality, accounting for approximately one quarter of neonatal deaths [13]. Effective resuscitation at birth, therefore, offers substantial potential to reduce mortality. Neonatal resuscitation has been shown to reduce intrapartum-related neonatal deaths by approximately 30% in health facilities, with high coverage potentially preventing up to 192,000 newborn deaths globally each year [14–16]. Nevertheless, the provision of effective resuscitation and neonatal life-saving interventions hinges upon the knowledge, skills and confidence of nurses, midwives, and other healthcare providers [8]. This poses a major challenge for low- and middle-income countries (LMICs), where workforce shortages, limited training, and inadequate equipment limit the provision of effective neonatal resuscitation [17].

Program sustainability is critical for lasting improvements in neonatal outcomes and requires continuous capacity building, self-sufficiency, and stakeholder engagement [18]. For this review, sustainability refers to the extent to which training programs maintain impact on provider competencies and clinical outcomes beyond the initial implementation period and are integrated into existing health system structures.

### Existing neonatal care training programs

Several initiatives have been implemented to improve healthcare workers’ knowledge and skills in neonatal resuscitation in LMICs. Building on the WHO Essential Newborn Care program, the American Academy of Paediatrics launched Helping Babies Breathe in 2010. HBB was later incorporated into the Helping Mothers and Babies Survive initiative in 2015 [19–23]. Several LMICs also implemented their own adaptations tailored to their health system contexts [24–26]. These evidence-based training programs typically use simulation-based learning, practical hands-on experience and refresher training sessions to maintain and strengthen the competencies of healthcare workers [27–29]. A train-the-trainer model is frequently used, in which local health workers serve as master trainers to strengthen local capacity and ensure training is culturally and contextually appropriate [29]. Local adaptation is a key factor influencing the effectiveness and long-term sustainability of these programs [29].

Earlier systematic reviews and meta-analyses evaluated the effectiveness of the Helping Babies Breathe program in LMICs, focusing on its impact on neonatal mortality and morbidity [30] and on resuscitation care practices, such as bag-and-mask ventilation at birth [31]. Both were confined to pre-pandemic literature and did not address program sustainability or changes in training delivery. A more recent review synthesised the effectiveness, cost-effectiveness, and scale-up of neonatal resuscitation training programs in LMICs, with searches run to August 2022 [32]. The present review extends beyond these: its search window reaches January 2025 (capturing evidence published from 2020 onward), and it adds an explicit focus on program sustainability, implementation characteristics, and the virtual and hybrid delivery modalities that emerged during COVID-19, none of which the earlier reviews examined, although the search by Sihota et al. [32] did overlap the early pandemic period. However, the emergence of COVID-19 in 2020 disrupted healthcare training and service delivery systems globally [33–35]. A reduction in neonatal resuscitation training sessions was observed during this period [36]. Contributing factors included social distancing requirements, restricted travel for trainers, and limited access to training facilities and equipment, such as simulation mannequins [37,38]. In response, virtual platforms and hybrid training models became widely used [33], representing a shift from traditional face-to-face neonatal resuscitation training approaches [39]. Given the change in the training environment and rapid emergence of new delivery modalities since 2020, a contemporary synthesis is warranted to assess whether these adaptations have preserved or extended the effectiveness of neonatal resuscitation training in LMICs.

This systematic review had two objectives: (1) to evaluate the effectiveness of neonatal resuscitation training programs on healthcare provider competency outcomes and neonatal mortality in LMICs; and (2) to examine the sustainability and implementation characteristics of these programs. Both objectives were addressed using studies published between January 2020 and January 2025, in order to capture the most contemporary evidence, including pandemic-related training adaptations, relevant to progress toward Sustainable Development Goal 3.2 (SDG 3.2) targets by 2030.

## Methods

### Protocol

The protocol for this systematic review has been previously registered and is searchable in PROSPERO under registration number CRD42024623116. This review was conducted and reported in accordance with the Preferred Reporting Items for Systematic Reviews and Meta-Analyses (PRISMA) 2020 guidelines [40].

### Eligibility criteria

The review included primary research published in English from January 2020 to January 2025 evaluating formalised neonatal resuscitation programs, defined as programs with a defined curriculum, learning objectives, and standardised assessment components (including Helping Babies Breathe [HBB], Neonatal Resuscitation Program [NRP], and similar approaches), for healthcare providers (nurses, midwives, doctors, community health workers) in LMICs, reflecting the multidisciplinary task-sharing common in these settings. Experimental and observational studies across hospital, primary care, and community settings were eligible. Outcomes included program effectiveness (knowledge acquisition and retention, skill development, and clinical practice change), institutional (facility-based) neonatal mortality rates, where reported, and sustainability indicators (defined as continued program delivery beyond initial implementation, retention of trained providers, ongoing supervision and mentorship, sustained clinical practice change, and institutional embedding within routine service delivery); studies reporting only proxy outcomes were included, given the limited availability of mortality data in LMICs settings. Full inclusion and exclusion criteria are presented in Table 1.

**Table 1.** Inclusion and exclusion criteria.

Criteria	Inclusion criteria	Exclusion criteria
General Criteria	English language publications published from January 2020 to January 2025	Non-English publications
Population	Healthcare providers involved in neonatal care, doctors, nurses, community health workers, working in low- and middle-income countries (as defined by World Bank classification) in any healthcare setting (hospitals, clinics, community centres)	Health students (nursing, medical and midwifery students) not directly involved in healthcare delivery; healthcare providers working exclusively in high-income countries; and non-healthcare workers
Intervention	Neonatal resuscitation training programs, including skills-based workshops, simulation-based training, refresher training programs, train-the-trainer programs, online or e-learning programs, and hybrid training approaches	Education programs without specific neonatal resuscitation components; Training programs not focused on neonatal resuscitation; and theoretical lectures without practical components
Comparator	Any comparator, no training, standard training methods, different durations or intensities of training, different types of neonatal resuscitation training, alternative educational approaches	N/A
Outcomes	Neonatal mortality rates, training program effectiveness measures, program sustainability metrics, knowledge and skills retention, provider confidence and competence, implementation challenges and solutions, changes in clinical practice, training program completion rates	Outcomes unrelated to neonatal resuscitation; outcomes without clear measurement criteria.
**Study Types**	Randomised controlled trials, non-randomised controlled trials, quasi-experimental studies, before-and-after studies, prospective and retrospective cohort studies, interrupted time series analysis, analytical cross-sectional studies, mixed-method studies with quantitative outcomes	Case reports, qualitative studies without quantitative outcomes, opinion pieces, editorials, letters to the editor, conference abstracts, unpublished theses/dissertations

### Search strategy

An experienced librarian assisted in developing the MEDLINE search strategy from the text words and index terms of relevant articles, which was then adapted for each of the five databases searched: MEDLINE (Ovid), PubMed, CINAHL (EBSCOhost), Embase, and Scopus. MEDLINE (Ovid) and PubMed were searched separately to maximise retrieval, with overlap addressed through deduplication. The search was limited to recent studies published within the last 5 years (January 2020–January 2025). The MEDLINE search strategy is available in Supplementary File 1 in the Online Supplementary Document.

### Study selection

All identified citations were collated and uploaded to EndNote and Covidence; deduplication was conducted sequentially in both programs, with manual verification of remaining duplicates. Titles, abstracts, and full texts were independently screened by two reviewers against predefined criteria, with reasons for exclusions recorded, and a third reviewer consulted to resolve disagreements. The selection process is documented in a PRISMA flow diagram.

### Data extraction and synthesis

Data extraction was conducted systematically using a standardised form encompassing study characteristics (authors, year, country, income classification, study design), population characteristics (healthcare providers, settings), intervention details (training program, delivery methods, duration, implementation strategy), and outcome measurements (mortality rates, knowledge and skills assessment, clinical practice indicators). Follow-up periods were categorised as immediate (0–4 weeks post-training), short-term (>4 weeks to 6 months), medium-term (>6 to 12 months), and long-term (>12 months). A descriptive synthesis was conducted following thematic groupings based on outcome categories (mortality, provider competency, implementation factors, and sustainability indicators). A meta-analysis was not conducted due to substantial heterogeneity among the included studies in intervention types, outcome measures, assessment tools, follow-up durations, and study designs.

### Methodological quality assessment

The methodological quality of included studies was assessed independently by two reviewers using the Mixed Methods Appraisal Tool (MMAT) [41], which provides design-specific criteria for randomised controlled trials, non-randomised studies, and mixed-methods studies. Discrepancies between reviewers were resolved through discussion until consensus was reached, and no studies were excluded based on methodological quality.

## Results

### Study inclusion

The search identified 715 records across Embase (*n* = 230), PubMed (*n* = 171), MEDLINE (Ovid) (*n* = 119), Scopus (*n* = 116), and CINAHL (*n* = 79). After removing 357 duplicates (manual *n* = 2; Covidence *n* = 355), 358 records were screened, and 273 were excluded at the title/abstract. Of 85 full-texts reviewed, 33 were excluded as conference or abstract-only (*n* = 13), inappropriate setting (*n* = 1), incorrect outcomes (*n* = 2), incorrect intervention (*n* = 4), or incorrect study design (*n* = 13). Fifty-two studies met the inclusion criteria, all conducted in LMICs (Figure 1).

**Figure 1.**
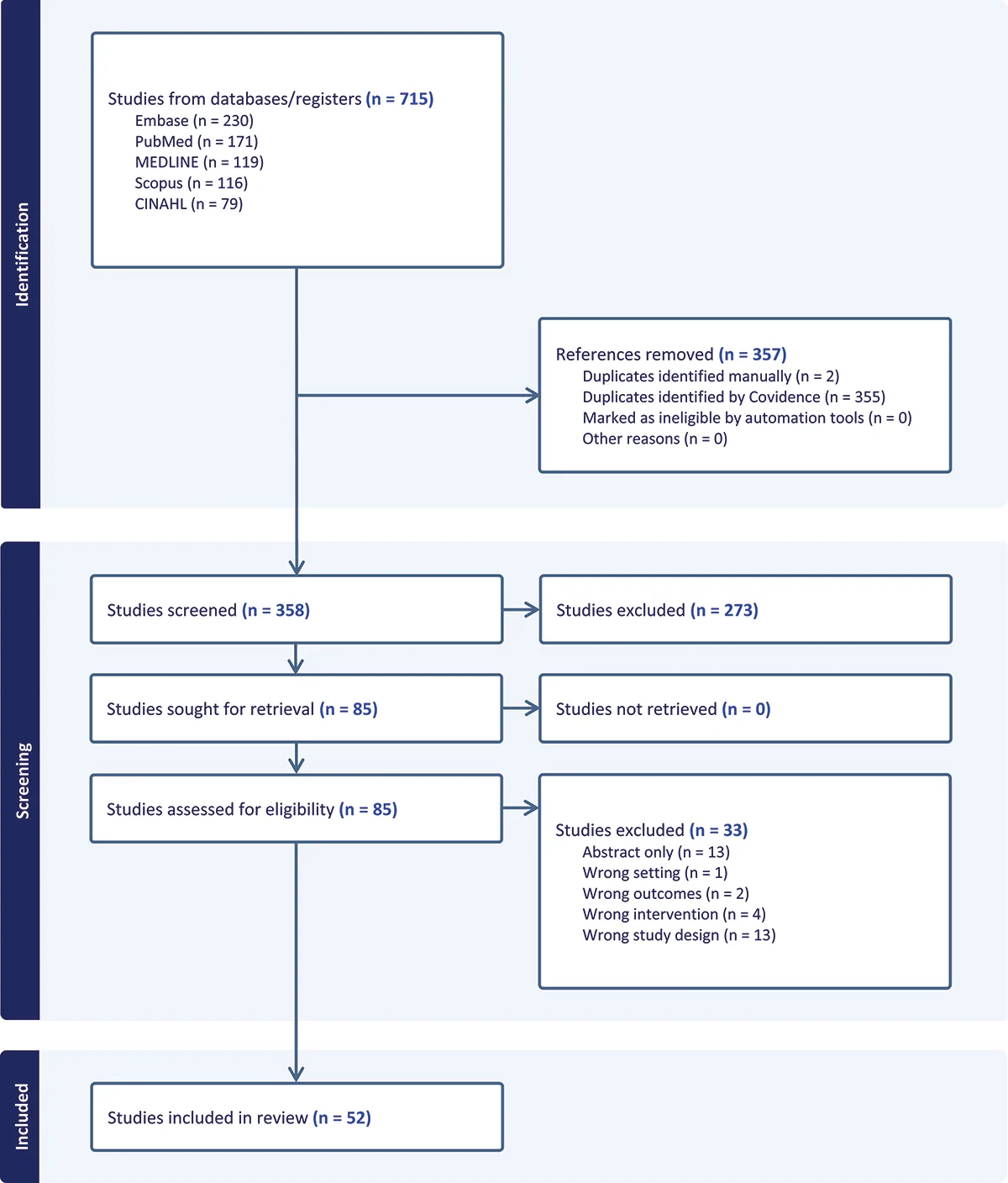
PRISMA flow diagram.

### Study characteristics

The 52 included studies represented 23 countries, with the majority conducted in Sub-Saharan Africa (*n* = 26, 50.0%) and South Asia (*n* = 12, 23.1%); the remaining studies were distributed across the Middle East, East Asia, Southeast Asia, the Americas, Oceania, and multi-country collaborations (counted once per study, with regions assigned by primary location). By income classification, 28 studies (53.8%) were conducted in lower-middle-income countries, 16 (30.8%) in low-income countries, and 8 (15.4%) in upper-middle-income countries (Table 2). Detailed descriptions of each study are available in Supplementary File 2.

**Table 2.** Study characteristics by geographic region and income classification of the included studies.

Region	Countries Represented (n)	Studies n (%)	Income Classification Distribution n (%)
Sub-Saharan Africa	9	26 (50.0)	Low: 15 (57.7) Lower-middle: 10 (38.5) Upper-middle: 1 (3.8)
South Asia	3	12 (23.1)	Lower-middle: 12 (100)
Middle East	3	4 (7.7)	Lower-middle: 1 (25) Upper-middle: 3 (75)
East Asia	1	1 (1.9)	Upper-middle: 1 (100)
Southeast Asia	1	1 (1.9)	Upper-middle: 1 (100)
Americas	2	2 (3.8)	Upper-middle: 2 (100)
Oceania	1	1 (1.9)	Lower-middle: 1 (100)
Multi-country	4	5 (9.6)	Low: 1 (20) Lower-middle: 4 (80)
**Total**	**23 countries**	**52 (100)**	**Low: 16 (30.8%)** **Lower-middle: 28 (53.8%)** **Upper-middle: 8 (15.4%)**

Notes:

Multi-country studies include collaborations involving Ghana [42,43], Nigeria and Kenya [37,44], and Madagascar [45].

Countries appearing in multi-country studies are also counted within their respective regional categories for the country count.

Income classifications are based on World Bank country classifications.

### Methodological quality of included studies

The methodological quality assessment revealed substantial heterogeneity across the 52 included studies (Table 3; Supplementary File 3). Overall, 15 studies (28.8%) had concerning risk of bias (moderate-to-high or high), while 37 (71.2%) were of acceptable methodological quality (low, low-to-moderate, or moderate). None of the eight RCTs had a high risk of bias; among the 42 non-randomised studies, 13 (31.0%) were rated at moderate-to-high or high risk of bias. Both mixed-methods studies were of moderate risk of bias.

**Table 3.** Methodological quality distribution by study design.

Study Design	n (%)	Low	Low-Mod	Moderate	Mod-High	High
RCTs	8 (15.4)	3 (37.5)	1 (12.5)	2 (25)	2 (25)	0 (0)
Non-randomised	42 (80.8)	2 (4.8)	6 (14.3)	21 (50)	11 (26.2)	2 (4.8)
Mixed methods	2 (3.8)	0 (0)	0 (0)	2 (100)	0 (0)	0 (0)
Total	52 (100)					

Study quality varied across the included studies. At the stronger end, three studies used rigorous randomisation or comprehensive confounder adjustment with high follow-up and blinded assessment [46–48], whereas several studies at the weaker end lacked control groups, baseline data, or blinding and relied on convenience sampling, precluding causal inference. Common methodological limitations included the absence of control groups (in approximately 80% of non-randomised studies), inadequate adjustment for confounders, unclear blinding of outcome assessors, high attrition, and limited follow-up duration; individual study-level assessments are provided in Table 3 and Supplementary File 3.

### Training program characteristics

Table 4 summarises the training programs across the included studies. Helping Babies Breathe (HBB) was the predominant training program, appearing in 31 studies (approximately 60%), either alone or in combination with other interventions, because studies often combined approaches; the categories are not mutually exclusive. Other studies evaluated country-specific, technology-supported, and specialised programs.

**Table 4.** Neonatal resuscitation training programs and delivery methods of the included studies.

Neonatal Resuscitation Training Program	Features	Studies n (%)
Helping Babies Breathe (HBB)	Basic resuscitation skills, simulation	15 (28.8)
Modified/Enhanced HBB	HBB + Quality Improvement, mentorship, Low-Dose High Frequency (LDHF), and equipment	16 (30.8)
Country-specific programs	Country-specific protocols (e.g. Brazil NRP, Ghana MEBCI, Malaysia NRP, India NSSK)	11 (21.2)
Technology-enhanced programs	Mobile app integration, VR simulation, eHBB	5 (9.6)
Other specialised programs	EENC, PRONTO, NeoLis, WHO ETAT+, etc	5 (9.6)

Note: Some studies appear in multiple categories because combined approaches were used.

### Healthcare provider competency outcomes

Knowledge and skill outcomes were reported in 37 studies (71.2%), assessed using standardised tools within training program packages (e.g. HBB knowledge checks, bag-and-mask ventilation skills assessments, Objective Structured Clinical Examinations) [37,42–47,49–77]. All studies reporting an immediate post-training comparison found improvement in at least one knowledge or skills outcome.

However, retention declined over time. Of the 37 studies reporting competency outcomes, 22 (59.5%) included follow-up assessments beyond the immediate post-training period: 8 at short-term (>4 weeks to 6 months), 9 at medium-term (>6 to 12 months), and 5 at long-term (>12 months). Among studies with follow-up data, the proportion of studies reporting maintained improvement decreased at longer intervals. At short-term follow-up, approximately 75% of studies reporting knowledge outcomes and 70% of those reporting skills outcomes demonstrated retained improvement above baseline levels [49,50,52,53,59,62,69,77]. At long-term follow-up (>12 months), these proportions declined to approximately 60% for knowledge and 55% for skills [46,60,61,74,75].

Studies without ongoing support (refresher training, mentorship visits, or low-dose high-frequency practice) demonstrated substantial skill decay across five studies [49,50,62,74,75]. At the longest follow-up assessment reported by each study, ranging up to 12 months post-training, knowledge scores declined by approximately 7% to 28%, and skills by approximately 4% to 35%, with all decreases statistically significant. Because follow-up did not exceed 12 months in any study, these findings represent short- and medium-term retention outcomes, as long-term follow-up was defined as more than 12 months. Individual study-level results are reported in Supplementary File 2.

Conversely, programs implementing LDHF or supportive-supervision approaches demonstrated superior retention across six studies [46,53,69,72,76,77]. Interventions combining supportive supervision, birth-record documentation, peer learning with mentorship, video-debriefing, or embedded quality-improvement tools maintained or improved knowledge and skill scores at medium-term follow-up (>6 to 12 months post-training). Individual study-level results are reported in Supplementary File 2.

### Neonatal mortality outcomes

Twenty-two studies (42.3%) reported institutional (facility-based) mortality outcomes, and 16 (72.7%) demonstrated a statistically significant reduction in at least one mortality indicator. Six studies specifically reported asphyxia-related mortality as a distinct outcome (rather than all-cause neonatal mortality) and all demonstrated significant reductions [76,78–82], providing focused evidence for the effect of training on the primary target condition. When disaggregated by outcome type, significant reductions were observed for early neonatal mortality in 13 of 19 studies (68.4%), fresh stillbirths in 10 of 17 studies (58.8%), and perinatal mortality in 11 of 15 studies (73.3%).

The 16 studies reporting significant mortality reductions varied in magnitude and outcome type. Reductions in neonatal or perinatal mortality ranged from approximately 27% to 72% [76,78–87], with additional significant improvements documented in fresh stillbirth rates and 24-hour mortality. Reductions were most pronounced in programs combining training with multi-component implementation (quality improvement, mentorship, and equipment provision) at scale, though causal attribution remains limited by the observational design of most studies. Individual study-level effect sizes are reported in Supplementary File 2.

Six studies reported no statistically significant mortality reduction despite improvements in provider competencies [63,71,72,88–90]. These null results were observed across diverse settings (Tanzania, India, Pakistan, Congo) and typically reflected small sample sizes, short follow-up periods, or contextual factors that may have limited detection of a mortality effect; individual study details are provided in Supplementary File 2.

### Implementation strategies and effectiveness

Multi-component interventions integrating training, mentorship, quality improvement, and equipment provision achieved the highest proportion of significant mortality reductions: among 11 such studies with mortality data [48,65,76,78,80,83–88]. Eight (73%) reported significant reductions, compared with approximately 50% of studies evaluating training-only interventions [42,72,91,92]. These proportions are derived from narrative classification of study results and were not statistically pooled or formally compared.

Local champion development was associated with improved participation and training frequency in the included studies; incorporating local motivators increased staff participation, monthly training frequency, and training quality in one Tanzanian program [90,93]. Systematic tiered training at national, regional, and district levels, with embedded quality improvement, achieved high pass rates for HBB knowledge and skills in Ghana [43].

Technology-enhanced approaches showed promise but variable effectiveness. Mobile applications supporting HBB were associated with improved skill retention and reduced birth asphyxia rates [81] and with improvements in neonatal outcomes and mortality [82]. Mobile virtual reality simulation improved specific skills, though with low overall pass rates [44], while virtual reality interventions showed no significant advantage over video-based or standard training [37].

### Sustainability: barriers and facilitators

No studies employed validated sustainability assessment tools such as the NHS Sustainability Model, the Program Sustainability Assessment Tool (PSAT), or the Clinical Sustainability Assessment Tool (CSAT) [94–96], a notable evidence gap given the centrality of sustainability to this review’s objectives. Sustainability was instead inferred from long-term outcome follow-up, program continuation reports, and qualitative descriptions of barriers and facilitators. Staff turnover was the most frequently cited barrier, reported across sub-Saharan African and South Asian settings, followed by resource constraints, particularly equipment unavailability. Technology and infrastructure barriers emerged during COVID-19, when poor internet connectivity and unfamiliarity with digital tools limited the use of hybrid and video-based delivery. Barriers reported by individual studies are detailed in Supplementary File 2.

Reported facilitators clustered around three themes: leadership and governance support, including government-led management and policy backing; local ownership, including institutional leadership and investment by local officials; and the integration of training with mentorship and quality improvement, which was associated with better outcomes across diverse contexts. The train-the-trainer model was described as a cost-effective means of scaling programs, and continuous learning environments with simulation-based methods were associated with improved program performance. Facilitators reported by individual studies are detailed in Supplementary File 2.

### COVID-19 impact and program adaptations

The COVID-19 pandemic significantly disrupted neonatal resuscitation training programs. Face-to-face sessions were suspended in four studies [45,83,84,87], with some facilities experiencing a complete cessation of training for up to 6 months [87]; mentorship and on-site coaching visits were curtailed [83]. Programs adapted through hybrid training models that combined limited in-person sessions with virtual components [84] and virtual platforms supporting live video streams and small-group skill practice, enabling continued training [45]. Traditional supervision was replaced by remote supervision through phone-based mentorship, messaging-based peer support groups, and virtual meetings [83,84,87].

COVID-adapted programs remained effective despite reduced face-to-face contact. Programs in Nepal that combined limited in-person training with phone- or video-based mentorship each reported significant reductions in fresh stillbirths and early (24-hour) neonatal mortality, with reductions of approximately 23% to 27% in the smallest of these programs and significant declines in most provinces during a national scale-up [83,84,87]. Individual study-level effect sizes are reported in Supplementary File 2.

## Discussion

The review of 52 studies across 23 LMICs demonstrates that structured training programs consistently improve provider knowledge and skills, with potential for mortality reduction when implemented with comprehensive system support. A consistent pattern emerged: multi-component interventions integrating training with mentorship, quality improvement, and equipment provision were associated with a higher proportion of significant reductions in mortality than standalone training approaches.

### Methodological quality and evidence interpretation

The predominance of non-randomised designs (80.8%) with limited control groups fundamentally constrains causal inference. All three RCTs at low risk of bias reported significant improvements in provider competencies [46,47,69], providing robust evidence of training’s effect on healthcare worker outcomes. However, the majority of mortality-reduction evidence derives from observational studies, where confounding from concurrent quality-improvement initiatives, equipment provision, or health-system strengthening cannot be excluded, as acknowledged by Brathwaite et al. [42] and Mayer et al. [85], whose significant improvements in mortality were reported alongside these concurrent activities.

The contrast between high-quality and lower-quality studies highlights important methodological considerations. For example, Stordal et al. [48], in one Tanzanian population, rated as low risk of bias through population-based sampling, comprehensive confounder adjustment, and a 5-year follow-up, demonstrated sustained mortality reduction with LDHF training. Conversely, studies with a higher risk of bias, such as Berg et al. [52] in one Ugandan setting with 41–42% attrition, Wilson et al. [77] in one setting with high attrition rates at the 6-month follow-up, or Brathwaite et al. [42] in one setting with non-comparable site designs and major confounding, require cautious interpretation despite positive findings, as their single-site design limits generalisability.

### Characteristics of effective programs

The evidence supports several characteristics distinguishing effective programs. The predominant use of HBB reflects its design for resource-limited settings, though the most effective programs extended beyond standard curricula to incorporate quality improvement, mentorship, and equipment provision. The higher proportion of studies reporting significant mortality reduction among multi-component interventions aligns with Sihota et al. [32] and Morris et al. [97] on the value of combined training-and-implementation packages; this narrative comparison may reflect confounding from unmeasured factors in non-randomised studies. Similarly, low-dose high-frequency (LDHF) or supportive-supervision approaches were associated with sustained competencies across six studies [46,53,69,72,76,77], consistent with evidence that post-training skill decline can be attenuated through low-dose, high-frequency refresher training.

Health system integration appears essential for achieving population-level impact. National programs in Nepal and Brazil, both integrating training with policy support and systematic scale-up, achieved substantial reductions in mortality. This suggests that vertical, project-based implementations may be insufficient for achieving sustained impact, supporting the integration of training programs within healthcare delivery systems.

### Effectiveness on mortality outcomes

Effectiveness in reducing institutional (facility-based) mortality was mixed: most studies reporting mortality demonstrated a significant decrease in at least one indicator. Still, the magnitude varied, and approximately one-quarter showed no statistically significant reductions. This variability is consistent with the findings of Versantvoort et al. [98], who reviewed seven studies, which identified only moderate evidence of reductions in intrapartum-related stillbirths and 24-hour neonatal mortality following the implementation of HBB.

The six studies that reported asphyxia-related mortality as a distinct outcome (rather than all-cause neonatal mortality) all demonstrated significant reductions, providing focused evidence for training’s effect on the primary target condition. This is consistent with Agudelo-Perez et al. [30], who reported significant asphyxia-related mortality reductions following HBB implementation. Effectiveness varied by context, with stronger health infrastructures and policy support associated with better outcomes. This is consistent with Morris et al. [97], where staff turnover and time constraints were identified as key barriers prevalent in limited-resource settings. Notably, no included study reported near-miss indicators, such as the proportion of asphyxiated newborns requiring intubation after unsuccessful bag-and-mask ventilation. Nine studies (17.3%) reported related escalation or severe-morbidity outcomes, including severe asphyxia or hypoxic-ischaemic encephalopathy, neonatal intensive care admission or transfer, and sick-newborn transfer used as a morbidity proxy. Still, definitions were inconsistent and not amenable to synthesis. Such indicators would capture the immediate effect of resuscitation on the target condition and complement mortality endpoints that are prone to under-detection in small or short-duration studies.

### Sustainability features and challenges

Sustainability is a critical determinant of long-term effectiveness, yet only 19 studies (36.5%) reported outcomes beyond the immediate post-training period, and none used a validated sustainability assessment tool. This limits the ability to draw robust conclusions about the durability of program impact.

Among studies with extended follow-up, comprehensive support systems were consistently associated with improved sustainability, with the development of local champions being particularly effective. In Tanzania, Vadla et al. [93] reported that designated champions increased participation and training frequency in one district-level program. Complementary evidence from peer learning with mentorship, structured supportive supervision, and district-level mentoring at scale, together with virtually mentored training and the adaptive context-specific training framework advocated for LMIC settings, converges on the value of local champion and mentorship approaches [45,46,76,77,99]. However, these findings derive predominantly from single-site studies and require confirmation through comparative evaluations.

Staff turnover constitutes the most pervasive barrier, creating continuous training cycles that strain constrained resources [57,91]. Equipment unavailability persists even in programs with government support [35,88,91], reflecting deeper supply-chain and maintenance limitations in low-resource facilities.

These findings align with the broader implementation literature, which demonstrates that sustainability measurement remains critically underdeveloped in neonatal health interventions [32,97,98]. Van Heerden et al. [100] reported that action research in South Africa achieved sustained improvements in staff competency and reduced neonatal mortality over 2 years. In contrast, Eriksson et al. [101] found that, in Vietnam’s NeoKIP trial, gains were not sustained 3 years after implementation, owing to limited stakeholder engagement, absent handover planning, and weak integration into health-system structures. Arul et al. [102] reported that sustaining simulation programs in a high-income setting required champion teams with protected time and integration into mandatory competency requirements, yet encountered barriers similar to those in LMICs, including staff turnover and competing priorities.

### COVID-19 adaptation: lessons for sustainability

The pandemic demonstrated program resilience and adaptability across neonatal health services [34–36]. Hybrid models combining virtual and in-person elements emerged as viable alternatives for maintaining program delivery when conventional approaches were disrupted. For example, Dhungana et al. [84] reported reduced 24-hour neonatal mortality following the implementation of phone- and video-based mentorship in Nepal, although the effectiveness of this approach was constrained in areas with poor internet connectivity. Similarly, reviews by Boutros et al. [33] and Khurshid et al. [39] found that simulation training, e-learning, and virtual quality-improvement adaptation were able to sustain, and in some cases enhance, health-worker education during the pandemic. While these reviews were not specific to neonatal resuscitation, they provide evidence that virtual and hybrid approaches can support workforce development when in-person training is limited. Systematic reviews of e-learning in nursing and midwifery [103] and of digital learning in nursing education support the effectiveness of these approaches while identifying persistent challenges related to access to technology, internet connectivity, and constraints on clinical skill practice. Virtual components also helped address participation constraints at remote facilities, suggesting that hybrid delivery may extend program reach while maintaining continuity.

In the specific context of newborn resuscitation, a scoping review of digital training technologies in LMICs found e-learning platforms and mobile applications to be feasible and effective for building provider competency, but identified limited evidence regarding the resources needed to sustain them and their long-term impact [104]. Consistent with this, an online obstetric and neonatal emergency simulation for health-professional students in India supported effective interprofessional learning during the pandemic, while noting that reliable internet and digital infrastructure are not uniformly available across LMICs [105]. A global systematic review of health-worker education during COVID-19 similarly documented severe disruption alongside broad learner acceptance of online and blended formats [106]. Limitations in practical experience and interpersonal interaction across maternal and perinatal services have been reported by Kotlar et al. [107]. The enforced innovation revealed that hybrid approaches can enhance program coverage and sustainability in resource-constrained settings, providing a foundation for future resilience strategies beyond pandemic contexts.

## Limitations

### Limitations of included studies

The predominance of non-randomised designs with no control groups (approximately 80% of observational studies) precludes definitive causal attribution of observed improvements to training interventions rather than to secular trends or co-interventions, and inadequate adjustment for confounders often prevents the isolation of training effects from concurrent quality improvement, equipment provision, or policy changes. Substantial heterogeneity in interventions, outcome measures, assessment tools, and follow-up periods further limits cross-study comparability. These challenges are well recognised in non-randomised intervention studies, where residual confounding remains a key threat to causal inference [108]. Furthermore, clinical and methodological heterogeneity across studies limits the validity of cross-study comparisons and warrants cautious interpretation of the findings [109].

High attrition rates may introduce selection bias, and variable intervention fidelity complicates interpretation of the synthesis findings. The predominance of immediate post-training assessments limits understanding of sustainability, particularly given documented skill decay. Many studies also relied on proxy outcomes (knowledge and skills) rather than clinical outcomes, limiting the ability to determine whether competency gains translated into sustained improvements in neonatal outcomes. Near-miss indicators (for example, the proportion of neonatal asphyxia cases requiring intubation after unsuccessful bag-and-mask ventilation) were not reported in the 52 included studies, and the 9 studies reporting related escalation or severe-morbidity outcomes used inconsistent definitions, despite these outcomes representing an important measure of neonatal resuscitation program effect on the primary target condition.

### Limitations of this review

Restrictions on English publications may have excluded relevant non-English studies from LMICs, introducing selection bias. Substantial heterogeneity precluded meta-analysis. Publication bias toward positive findings may inflate the apparent effectiveness of interventions, and the focus on major indexed databases without extensive grey literature searches may have excluded program evaluations not published in peer-reviewed journals.

The MMAT ‘Can’t tell’ category was used when information was insufficient, potentially underestimating quality when information was unreported rather than absent. Additionally, comparative statements regarding the relative effectiveness of multi-component versus training-only interventions are based on narrative synthesis and should not be interpreted as evidence of causal superiority, as formal subgroup analysis or meta-regression was not conducted. Finally, the review relied on published study reports; incomplete reporting in primary studies may have limited the ability to fully assess methodological quality and implementation characteristics.

## Implications

### Implications for policy and practice

Despite methodological limitations, consistent findings across diverse contexts support three priority actions for policymakers: (i) invest in comprehensive, system-integrated training programs that enhance provider competencies; (ii) prioritise multi-component approaches that integrate training with mentorship, quality improvement, and equipment provision over standalone training; and (iii) embed training within health system integration, as exemplified by the national programs in Nepal and Brazil, over vertical, project-based implementations.

### Implications for program design and sustainability evaluation

Standalone training is insufficient for sustainable outcomes. Effective and sustainable programs should include: (i) integration of quality improvement initiatives, low-dose high-frequency training, refresher sessions, mentoring, local leadership development, and simulation-based practice; (ii) alignment with national policies rather than vertical implementation; (iii) local champion development to enhance participation and training frequency; (iv) flexibility to local contexts, including infrastructure constraints; (v) built-in monitoring of process and outcome indicators over extended periods; and (vi) standardised sustainability assessment frameworks, clear handover criteria for local institutions, and capacity building for ongoing quality improvement beyond initial training.

### Implications for research

Future research should prioritise: (i) rigorous trial designs with blinded outcome assessment, supplemented by observational studies with robust adjustment for concurrent interventions, health system changes, and participant characteristics; (ii) extended follow-up of at least 12–24 months with effective retention strategies to address the sustainability evidence gap; (iii) systematic documentation of implementation fidelity (attendance, adherence, dose received); (iv) standardised outcome measurement using validated tools, consistent mortality definitions, and near-miss outcomes such as intubation following unsuccessful bag-and-mask ventilation; and (v) development, validation, and application of a sustainability assessment framework tailored to neonatal resuscitation training in low- and middle-income countries. These priorities would strengthen evidence synthesis and conclusions about long-term effectiveness across diverse settings.

## Conclusions

Neonatal resuscitation training programs in LMICs consistently improve healthcare provider competencies and, when implemented with system support, are associated with reduced neonatal mortality. Multi-component, system-integrated approaches achieve stronger outcomes than standalone training, with important implications for global health policy. Although the predominance of non-randomised designs and substantial heterogeneity warrant cautious interpretation of mortality estimates, uniform competency improvements across all designs support continued investment in structured training programs.

Reducing neonatal mortality in LMICs requires evidence-based interventions characterised by sufficient intensity, duration, and system support. By addressing the sustainability challenges and methodological limitations identified in this review, and building on the characteristics of successful programs, future research and program implementation can strengthen the evidence base and accelerate progress toward the Sustainable Development Goal of ending preventable neonatal mortality by 2030.

## Supplementary Material

PRISMA_2020_checklist.docx

PRISMA_2020_abstract_checklist.docx

Supplementary file 3_Methodological Quality of Included Studies.docx

Supplementary file 2_Description of Included Studies.docx

Supplementary file 1_Search strategy.docx

## Data Availability

The supplementary materials supporting this systematic review (search strategy, characteristics of included studies, and methodological quality assessment) are openly available on Figshare at https://doi.org/10.6084/m9.figshare.32644173. The 52 studies synthesised in this review are all peer-reviewed publications cited in the reference list and accessible through their respective publishers. The review protocol is registered with PROSPERO (CRD42024623116).
