# Supplementary material for: Effectiveness and sustainability of neonatal resuscitation training programs in reducing neonatal mortality rates in low- and middle-income countries: a systematic review
Source: Glob Health Action. 2026 Nov 5;19(1):2757387. doi: 10.1080/16549716.2026.2757387 (PMC13757925; doi:10.1080/16549716.2026.2757387)
Supplement: Supplementary file 1 [file ZGHA_A_2757387_SM7804.docx]

**Supplementary file 1: Medline search strategies**

**Medline Search Strategy**

1. exp Resuscitation/
2. ((cardiac or cardio* or heart) adj3 massage*).tw.
3. OR/1-2
4. exp Infant, Newborn/
5. (neonat* or newborn* or "new born*" or infant*).tw.
6. OR/4-5
7. 3 AND 6
8. (resuscitat* adj3 (neonat* or newborn* or "new born*" or infant*)).tw.
9. ("helping babies breathe" or "helping babies survive" or "helping babies survive and thrive").tw.
10. ("essential newborn care" or "neonatal life support" or "birth asphyxia program*" or "managing newborn problems").tw.
11. "safe delivery app".tw.
12. ("navjaat shishu suraksha karyakram" or NSSK).tw.
13. ("emergency triage assessment and treatment" or ETAT or ETAT+).tw.
14. ("early essential newborn care" or EENC).tw.
15. ("basic emergency obstetric and newborn care" or BEmONC).tw.
16. ("comprehensive emergency obstetric and newborn care" or CEmONC).tw.
17. ("neonatal resuscitation program*" or NRP).tw.
18. ("making every baby count initiative" or MEBCI).tw.
19. ("all babies count initiative" or ABC).tw.
20. ("essential care for every baby" or ECEB).tw.
21. "helping babies survive and thrive".tw.
22. ("essential care for labor and birth" or "essential care for labour and birth" or "HMS ECL&B" or ECL&B).tw.
23. ("essential care for small babies" or ECSB).tw.
24. ("emergency obstetric and newborn care" or EmONC).tw.
25. ("integrated management of pregnancy and childbirth" or IMPAC).tw.
26. ("pregnancy, childbirth, postpartum and newborn care" or PCPNC).tw.
27. or/8-26
28. exp Education/ or exp Teaching/ or exp Inservice Training/ or exp Education, Medical, Continuing/ or exp Education, Nursing, Continuing/
29. (train* or educat* or teach* or learn* or instruct* or course* or workshop* or program* or mentor* or coach* or facilitat* or "competency based").tw.
30. (skill* adj3 (develop* or build* or enhance* or maintain*)).tw
31. (capacity adj3 build*).tw.
32. (simulation adj3 (train* or educat* or teach* or learn*)).tw.
33. (translat* adj3 "clinical practice").tw.
34. or/28-33
35. exp Developing Countries/
36. (("low income" or "middle income" or developing) adj3 (country or countries or nation* or population*)).af.
37. (LMIC or LMICs or "third world" or "resource-limited" or "resource-poor").af.
38. afghanistan/ or albania/ or algeria/ or angola/ or argentina/ or armenia/ or azerbaijan/ or bangladesh/ or belize/ or benin/ or bhutan/ or bolivia/ or borneo/ or "bosnia and herzegovina"/ or botswana/ or brazil/ or burkina faso/ or burundi/ or cabo verde/ or cambodia/ or cameroon/ or central african republic/ or chad/ or china/ or colombia/ or comoros/ or congo/ or costa rica/ or cote d'ivoire/ or cuba/ or "democratic people's republic of korea"/ or "democratic republic of the congo"/ or djibouti/ or dominica/ or dominican republic/ or ecuador/ or egypt/ or el salvador/ or equatorial guinea/ or eritrea/ or eswatini/ or ethiopia/ or fiji/ or gabon/ or gambia/ or "georgia (republic)"/ or ghana/ or grenada/ or guatemala/ or guinea/ or guinea-bissau/ or haiti/ or honduras/ or exp india/ or indonesia/ or iran/ or iraq/ or jamaica/ or jordan/ or kazakhstan/ or kenya/ or kyrgyzstan/ or laos/ or lebanon/ or lesotho/ or liberia/ or libya/ or madagascar/ or malawi/ or malaysia/ or maldives/ or mali/ or mauritius/ or exp melanesia/ or mexico/ or exp micronesia/ or moldova/ or mongolia/ or montenegro/ or morocco/ or mozambique/ or myanmar/ or namibia/ or nepal/ or nicaragua/ or niger/ or nigeria/ or pakistan/ or papua new guinea/ or paraguay/ or peru/ or philippines/ or "republic of belarus"/ or "republic of north macedonia"/ or rwanda/ or saint lucia/ or "saint vincent and the grenadines"/ or exp samoa/ or "sao tome and principe"/ or senegal/ or serbia/ or sierra leone/ or somalia/ or south africa/ or south sudan/ or sri lanka/ or sudan/ or suriname/ or syria/ or tanzania/ or thailand/ or timor-leste/ or togo/ or tonga/ or tunisia/ or turkey/ or turkmenistan/ or uganda/ or ukraine/ or uzbekistan/ or vanuatu/ or venezuela/ or vietnam/ or yemen/ or zambia/ or zimbabwe/
39. (Afghanistan or Albania or Algeria or Angola or Argentina or Armenia or Azerbaijan or Bangladesh or Belarus or Belize or Benin or Bhutan or Bolivia or Botswana or Bosnia or Brazil or "Burkina Faso" or Burundi or "Cabo Verde" or Cambodia or Cameroon or "Central African Republic" or Chad or China or Colombia or Comoros or Congo or "Costa Rica" or "Cote d'Ivoire" or "Ivory Coast" or Cuba or Djibouti or Dominica or "Dominican Republic" or Ecuador or Egypt or "El Salvador" or Eritrea or Eswatini or Ethiopia or Fiji or Gabon or Gambia or Georgia or Ghana or Grenada or Guatemala or Guinea or Haiti or Herzegovina or Honduras or India or Indonesia or Iran or Iraq or Jamaica or Jordan or Kazakhstan or Kenya or Kiribati or "Korea Democratic People's Republic" or "North Korea" or Kosovo or "Kyrgyz Republic" or Kyrgyzstan or "Lao PDR" or Laos or Lebanon or Lesotho or Liberia or Libya or Madagascar or Malawi or Malaysia or Maldives or Mali or "Marshall Islands" or Mauritania or Mauritius or Mexico or Micronesia or Moldova or Mongolia or Montenegro or Morocco or Mozambique or Myanmar or Namibia or Nepal or Nicaragua or Niger or Nigeria or "North Macedonia" or Pakistan or Paraguay or Peru or Philippines or Rwanda or Samoa or "Sao Tome and Principe" or Senegal or Serbia or "Sierra Leone" or "Solomon Islands" or Somalia or "South Africa" or "South Sudan" or "Sri Lanka" or "St. Lucia" or "St Lucia" or "Saint Lucia" or "St. Vincent and the Grenadines" or "St Vincent and the Grenadines" or "Saint Vincent and the Grenadines" or Sudan or Suriname or "Syrian Arab Republic" or Syria or Tajikistan or Tanzania or Thailand or "Timor-Leste" or Togo or Tonga or Tunisia or Turkey or Turkmenistan or Tuvalu or Uganda or Ukraine or Uzbekistan or Vanuatu or Venezuela or Vietnam or "Viet Nam" or "West Bank" or Gaza or Yemen or Zambia or Zimbabwe).af.
40. or/35-39
41. exp Program Evaluation/ or exp Treatment Outcome/ or exp Infant Mortality/ or exp Perinatal Death/ or exp Sustainability/ or exp Cost-Benefit Analysis/ or exp Health Care Costs/
42. (program* adj3 (evaluat* or effect* or success* or sustain* or implement*)).tw.
43. (outcome* or impact* or mortality or death* or survival or sustainab* or maintain* or long-term or implementation or characteris* or feature* or component* or attribute* or cost* or economic* or feasib* or affordab* or barrier* or facilitat* or challeng* or enabler* or "scale-up" or rollout or "roll-out" or disseminat*).tw.
44. (quality adj3 (improvement or assurance or control)).tw.
45. (knowledge adj3 (retention or transfer or maintenance)).tw.
46. ((translat* or implement* or integrat*) adj3 practice).tw.
47. or/41-46
48. exp Students, Health Occupations/ or exp Students, Medical/ or exp Students, Nursing/ or exp Students, Midwifery/
49. (student* adj3 (medical or nursing or healthcare or "health care" or midwi* or resident* or intern*)).tw.
50. or/48-49
51. 7 and 27 and 34 and 40 and 47
52. limit 51 to yr="2020-current"
53. limit 52 to English
54. 53 not 50
