# Supplementary material for: Effectiveness and sustainability of neonatal resuscitation training programs in reducing neonatal mortality rates in low- and middle-income countries: a systematic review
Source: Glob Health Action. 2026 Nov 5;19(1):2757387. doi: 10.1080/16549716.2026.2757387 (PMC13757925; doi:10.1080/16549716.2026.2757387)
Supplement: Supplementary file 2 [file ZGHA_A_2757387_SM7805.docx]

**Supplementary file 2: Description of Included Studies, n=52**

| **Author** | **Country (Income Group)** | **Aim** | **Study Design** | **Trained Population**  **(Settings)** | **Training Program Used** | **Partner** | **Training Delivery Methods** | **Duration of Program Implementation** | **Follow-up Data Collection Period** | **Outcome measures** | **Key Findings** | **Additional Information** |
| --- | --- | --- | --- | --- | --- | --- | --- | --- | --- | --- | --- | --- |
| Abou-Zamzam et al. (2023) | Madagascar  (Low-income) | To assess the viability of a virtually mentored and flipped classroom modification of Helping Babies Breathe (HBB) training | Cross-sectional study | 97 physicians, midwives, and nurses  (Community healthcare centres) | Helping Babies Breathe (HBB) | Johns Hopkins University School of Medicine, USA; Jhpiego Madagascar | Virtually mentored training via Zoom (live video stream of small groups, simulation-based practice with NeoNatalie mannequins, real-time feedback from remote master trainers). Compared traditional didactic and flipped classroom approaches. Independent training conducted by local trainers following virtual mentorship. | September 2021 – May 2022 | Continuous automated monitoring over 2-year study period | (1) Knowledge (written assessment), (2) Skills (Objective Structured Clinical Examination [OSCE]), (3) Self-reported confidence | (1) No significant difference in written assessment scores between virtually mentored and independent training groups (92.8% vs 91.5%, p=0.62). (2) Significantly higher OSCE scores in the independent training group compared with the virtually mentored group (97.3% vs 89.5%, p<0.001). Both traditional and flipped classroom models produced significant pre-to-post improvements in written assessments (p<0.001 and p<0.05, respectively). | COVID-19 pandemic-related adaptation. Virtual mentoring followed by successful independent training supports efficacy of virtual dissemination. No comparator group (pre-intervention comparison only). |
| Bameri et al. (2024) | Iran  (Upper-middle-income) | To investigate the impact of ongoing workshop training of the HBB programme on the retention of midwives' knowledge and skills | Quasi-experimental pre-post study | 61 midwives (53 met inclusion criteria; 38 completed follow-up)  (Hospital delivery and operating rooms) | Helping Babies Breathe (HBB) | Not reported | Workshop-based HBB training (3-hour sessions including lectures, slide presentations, interactive Q&A, group discussions, and hands-on exercises with manikins). Repeated workshops at 3, 5, and 6 months after initial training for reinforcement. | January 2022 – January 2023 | 6 months post-final workshop (12 months from initial training) | (1) Knowledge (18-item MCQ), (2) Skills (OSCE A [routine newborn care], OSCE B [neonatal resuscitation], OSCE BMV [bag-mask ventilation]) | (1) Significant improvement in knowledge from baseline to 6 months (p<0.05); knowledge maintained at 12 months with no significant decline from 6 months (p>0.05). (2) Significant improvement in OSCE A and B skills at 6 months compared with baseline (p<0.05); however, significant skill decay at 12 months compared with both baseline and 6-month scores (p<0.05). BMV skills declined at both 6 and 12 months. | Continuous education through repeated workshops maintained knowledge but skills declined without ongoing training. No comparator group (pre-intervention comparison only). |
| Baumgartner et al. (2021) | Uganda  (Low-income) | To estimate the impact of a clinical and management training programme on adherence to quality of care standards during labour and delivery, and on pre-discharge neonatal and maternal mortality | Quasi-experimental pre-post observational study | Midwives, nurses, clinical officers, and doctors at 6 facilities  (Faith-based, not-for-profit primary healthcare facilities) | LifeNet International's Maternal and Neonatal Health (MNH) training package (13 modules aligned with WHO and American Academy of Pediatrics [AAP] evidence-based guidelines) | LifeNet International (NGO) | On-site monthly didactic and hands-on training modules (lectures, video demonstrations, simulation practice including neonatal resuscitation on mannequin, condom tamponade assembly), quality assurance activities, management training, and medical record standardisation over 10 months. | August 2017 – May 2018 (10 months) | Immediately post-intervention only (with 28-day follow-up phone call for mortality data) | (1) Clinical quality adherence (17 quality of care indicators via direct clinical observation), (2) Pre-discharge neonatal mortality, (3) 28-day neonatal mortality, (4) Maternal mortality | Data from 263 deliveries (pre-intervention) and 321 deliveries (post-intervention). (1) Significant increase in adherence for handwashing (PR=4.79), delayed cord clamping (PR=2.48), and partograph documentation (PR=4.01). (2) Decreased pre-discharge neonatal mortality prevalence (0.027 to 0.009). (3) 28-day neonatal mortality decreased (0.042 to 0.016). Considerable variation across clinics. | Staff turnover may have negated some training effects. Direct clinical observation used as gold standard for quality measurement. No comparator group (pre-intervention comparison only). |
| Brathwaite et al. (2020) | Ghana  (Lower-middle-income) | To evaluate two neonatal resuscitation training and evaluation approaches at regional hospitals | Pre-post observational study | 412 healthcare providers (120 at NRP site; 292 at HBB/ECEB sites): midwives, neonatal nurses, theatre and recovery room nurses, nurse anaesthetists, physicians, senior nurses, and community health workers  (5 regional hospitals) | Two approaches compared: (1) Neonatal Resuscitation Program (NRP), modified for context; (2) Making Every Baby Count Initiative (MEBCI): Helping Babies Breathe (HBB), Essential Care for Every Baby (ECEB), and infection prevention training | Kybele Inc. (US-based non-profit); Ghana Health Service (GHS); PATH | NRP site: US-based instructors conducted real-time clinical observations, context-specific training (one-day 4-hour sessions), supportive supervision, bedside mentoring, and train-the-trainer. HBB/ECEB sites: 5-day off-site training (didactic presentations, hands-on manikin practice, role play), follow-up OSCE assessments at fixed intervals. | 2013 – 2018 (approximately 5 years across both approaches) | NRP: 6–9 months post-training (clinical observations and chart review). HBB/ECEB: 4–6 weeks, 5–6 months, and 12–13 months post-training (OSCE assessments) | (1) Knowledge (written pre/post-test), (2) Skills (real-time resuscitation observations [NRP site]; OSCE simulations [HBB/ECEB sites]), (3) Neonatal outcomes (fresh stillbirth [FSB] rate, institutional newborn death [IND] rate) | NRP site (GARH): Significant improvement in knowledge (p<0.01) and skills (p<0.01); significant decrease in FSB and IND rates (p<0.05). HBB/ECEB sites: OSCE pass rates exceeded 90% but follow-up participation decreased from 98% to 84% over 12 months; no significant improvement in FSB or IND rates. NRP results cannot be solely attributed to resuscitation training due to concurrent capacity-building activities. | High staff turnover and rotation hindered programme success. National programmes should tailor training to facility level. No comparator group (pre-intervention comparison only). |
| Briggs et al. (2021) | Nigeria  (Lower-middle-income) | To assess primary healthcare workers' retention of knowledge and skills in basic neonatal resuscitation | Prospective interventional pre-post study | 106 doctors and nurses/midwives across 28 primary healthcare centres  (Primary healthcare centres) | Neonatal Resuscitation Training (NRT), adapted from the AAP Neonatal Resuscitation Program (NRP) 6th edition | Not reported | One-day training (8 hours) with didactic lectures and hands-on simulation using bag and mask on NeoNatalie mannequin, small groups (trainer-to-trainee ratio 1:5). Equipment (bag-mask sets, reservoir bags, oxygen tubing) provided to facilities post-training. Refresher training conducted at 6-month assessment. | April 2018 – October 2018 | 3 months (subset of 30 participants) and 6 months (all participants) | (1) Knowledge (18-item MCQ; adequate defined as ≥80%), (2) Skills (OSCE; adequate defined as ≥80% plus completion of 5 critical steps) | (1) Significant increase in knowledge immediately post-training (35.2% to 81.5%, p=0.0001); significant decrease at 3 months (55.4%, p=0.0001) and 6 months (55.8%, p=0.0001). (2) Significant increase in skills immediately (21.4% to 87.4%, p=0.0001); significant decrease at 3 months (59.1%, p=0.0001) and 6 months (60.4%, p=0.0001). Following refresher training at 6 months, knowledge and skills scores increased to 94.9% and 96.0% respectively (p=0.0001). Approximately 90% of participants had inadequate knowledge and skills by 6 months without retraining. | One-off training insufficient; regular retraining (at least every 3–6 months) recommended. No comparator group (pre-intervention comparison only). |
| Cetinkaya et al. (2022) | Turkey  (Upper-middle-income) | To investigate the knowledge level of nurses and midwives who completed Neonatal Resuscitation Program (NRP) practitioner training and the effect of time since training on knowledge | Cross-sectional survey | 105 nurses and midwives  (Hospital neonatal intensive care units [NICU], gynaecology, and maternity wards) | Neonatal Resuscitation Program (NRP) practitioner training course | Not reported (national programme in collaboration with Turkey Ministry of Health and Neonatology Association) | NRP practitioner training course (visual presentations, training book, practical training on model). Part of national in-service training programme arranged 12 times in Adana Province. | Not reported (data collected January 2016; participants trained at varying prior intervals) | Cross-sectional assessment at single time point; participants stratified by time since NRP course (0–6 months, 6 months–1 year, 1–3 years, 4–5 years) | (1) Knowledge (30-item questionnaire on NRP content) | Mean knowledge score was 23.06 ± 3.47 out of 30 (intermediate level). No statistically significant difference between time since NRP course and mean knowledge score (p=0.33). Highest scores among those trained 0–6 months ago (23.9 ± 2.8); lowest among those trained 4–5 years ago (22.2 ± 3.4). Significant association between NRP course attendance time and age (p<0.05) and working years (p<0.05). | Cross-sectional design; no pre-training baseline measurement. Knowledge scores were reasonable despite most participants (37%) having been trained 4–5 years prior. Authors recommend systematic in-service training repetition. No comparator group. |
| Chalise et al. (2022) | Nepal  (Lower-middle-income) | To describe the implementation of a newborn resuscitation training, scale-up, and skill retention programme, and report changes in perinatal outcomes | Prospective before-after (2 phases) clinical observational study | 88 facility-based trainers (Training of Trainers [ToT]) cascading to 231 physicians, nurses, and midwives across 20 facilities  (Public and private hospitals) | Helping Babies Breathe (HBB) training through the Safa Sunaulo Nepal (SSN) programme | Latter-day Saint Charities (LDS Charities); National Health Training Center (NHTC), Nepal | Three-phase programme: (1) ToT phase with standardised HBB curriculum; (2) Scale-up and skill retention phase with facility-based cascade training, low-dose high-frequency (LDHF) practice sessions, review meetings, refresher training, on-site coaching by external mentor, and monitoring of key indicators; (3) Post scale-up phase with facility-based trainers continuing independently. | October 2020 – November 2021 (14 months) | Prospective monitoring over 14 months; baseline (first 2 months) vs endline (last 2 months) comparison | (1) Neonatal mortality (<24 hours and >24 hours), (2) Intrapartum stillbirths, (3) Newborn morbidity (sick newborns transferred from maternity unit as proxy), (4) Bag-mask ventilation utilisation | Data from 68,435 vaginal and 9,253 caesarean deliveries. (1) Significant 56% decrease in <24-hour neonatal deaths (p<0.001); no significant change in >24-hour deaths (p=0.067). (2) Significant 57% decrease in intrapartum stillbirths (p<0.001). (3) Significant 33% decrease in sick newborns transferred from maternity unit (p<0.001). Greater impact observed in primary and private hospitals. | Low-cost, scalable model using a single external mentor for 20 facilities. COVID-19 pandemic disrupted implementation consistency. No comparator group (pre-intervention comparison only). |
| Chan et al. (2023) | Uganda  (Low-income) | To assess whether a mobile app, HBB Prompt, developed with user-centred design, helps improve skills and knowledge retention after HBB training | Comparative study | 29 healthcare workers (HCWs): 17 intervention, 12 control; primarily midwives.  (Hospital) | HBB training | Grand Challenges Canada | Control: Standard in-person HBB training session (Small group discussion, Peer-to-peer facilitation, Simulation using NeoNatalie simulators, Debriefing sessions), practice skills at least once per shift and record it in logbook. Intervention: Standard in-person HBB training session + HBB Prompt/Mobile app with 4 components: Training mode (videos), Simulation mode (practice scenarios, Quizzes/Knowledge check, Scoreboard/Dashboard | April – October 2019 | 6 months (reduced due to COVID-19, initially planned for 1 year) | (1) Skills retention (OSCE B), (2) Knowledge (HBB 2.0 knowledge check), (3) Neonatal outcomes (birth asphyxia rates and fresh stillbirth rates) | (1) Significant difference in skills retention with less decay in intervention group (Intervention: median OSCE B score decline of -3 points (IQR -5 to -1); Control: median score decline of -8 points (IQR -11 to -6); p=0.02). (2) No significant difference in knowledge retention (p=0.68). (3) Significant decrease in birth asphyxia rates in intervention site (p=0.0004); non-significant downward trend in fresh stillbirth rates. | COVID-19 forced early termination (6 months instead of planned 12 months). Low app usage despite positive findings. Comparator: control hospital without access to HBB Prompt (standard HBB training only). |
| Chaulagain et al. (2021a) | Nepal  (Lower-middle-income) | To evaluate the effect of a scaled-up quality improvement (QI) intervention on uptake and retention of neonatal resuscitation knowledge and skills in simulated settings | Pre-post study | 380 Nurses, Auxiliary Nurse-Midwives (ANMs), Doctors across 12 hospitals.  (Hospitals) | HBB training and QI intervention | Vetenskapsrådet (Swedish Research Council), Laerdal Foundation for Acute Medicine (Norway), Einhorn Family Foundation (Sweden) | Selection of QI facilitators and external mentors, on-site basic training (3-day package), provision of QI tools (HBB job aid, self-assessment checklist, HBB mannequin set, scoreboards, weekly PDSA review meeting notes), weekly PDSA meetings, daily skill checks using mannequin, scoreboard with major indicators on neonatal resuscitation was installed, and refresher training (6 months after) | July 2017-Dec 2018 | 6 months | (1) Knowledge (17-item MCQ), (2) Skills (OSCE B) | (1) Significant increase in knowledge mean scores before and after training (14.12, SD=2.69 to 15.91, SD=2.08, p<0.001); significant decrease after 6 months (15.91 to 15.33, SD=2.15, p<0.001), however still above the set standard (>14.0). (2) Overall skill score during basic training (16.98, SD=1.79) deteriorated over time to 16.44, SD=1.99 during refresher training (p<0.001), however still above the set standard (>14.0). | Regular HBB training practice and QI tools improved knowledge and skills irrespective of the background characteristics of the health workers. No comparator group (pre-intervention comparison only). |
| Chaulagain et al. (2021b) | Nepal  (Lower-middle-income) | To evaluate the effect of a scaled-up QI intervention package on the performance of health workers on basic neonatal resuscitation care among non-crying infants | Prospective observational study | Paediatricians, medical officers, and nurses across 4 high-volume public hospitals  (Hospitals) | Quality Improvement intervention package | Vetenskapsrådet (Swedish Research Council), Laerdal Foundation for Acute Medicine (Norway), Einhorn Family Foundation (Sweden) | Master Training of Trainers on QI package, QI facilitators, readiness and availability perinatal care services assessment, bottleneck analysis workshop, QI package training, QI package (HBB job aid, self-assessment checklists, HBB mannequin set for the skill check, scoreboards, and weekly PDSA review meeting notes), refresher course six months after. | July 2017-Oct 2018 | Immediately post-training only | (1) Additional stimulation, (2) Clearing the airway, (3) Initiation of bag-and-mask ventilation (BMV), (4) Initiation of BMV within 1 min, (5) Time taken to initiate first ventilation, (6) Appropriate ventilation rate | Data collected from 3031 non-crying infants after birth. (1) A lower proportion of additional stimulation to non-crying infants (aOR 0.18; 95% CI 0.13–0.26). (2) The airway clearing proportion increased among non-crying infants (aOR 1.23; 95% CI 1.03–1.46). (3) Compared to the control, the proportion of non-crying infants initiated on BMV was higher (aOR 1.28, 95% CI 1.04–1.57). (4) The cumulative median time to initiate ventilation during the intervention was 39.46 s less compared to the baseline | First multi-centre large-scale study to assess effectiveness of scaled-up QI interventions on neonatal resuscitation performance in Nepal. QI intervention introduced in existing hospital set-up without structural modifications. Data collectors could not observe all deliveries; only 62% of total deliveries observed during study period. No comparator group (pre-intervention comparison only). |
| Chinbuah et al. (2020) | Ghana  (Lower-middle-income) | To describe the design and implementation of MEBCI’s innovative training approach to improve district-level health workers’ knowledge and skills in newborn resuscitation, essential newborn care, and newborn infection prevention | Programmatic training evaluation study | 3,688 Healthcare providers (Paediatrician/ Obstetrician,  Anaesthetist, Midwife, Medical Officers, General Nurses, PN/CN/EmN/PON, EnN/HA/WA, CHN and PHN, MA/PA, RHD/DHD from 151 facilities.  (Health facilities) | Making Every Baby Count Initiative (MEBCI): Helping Babies Breathe (HBB), Essential Care for Every Baby (ECEB), and Infection Prevention (IP) training | PATH; Kybele Inc.; Ghana Health Service (GHS); Children’s Investment Fund Foundation (CIFF) | The program was embedded within the existing public healthcare delivery system at the national, regional, and district levels consisting of six steps: 1) national level planning (developing training package, procuring the newborn equipment, front-line trainers trained), 2) selection and orientation of national experts, 3) selection and training of Master Trainers, 4)Selection and training of Regional Trainers, 5) implementation of HW trainings, and 6) implementation of supportive supervisory follow-up visits of the trained. HWs. | June 2015-July 2017 | Immediately post-training only | (1) Knowledge, (2) Skills | (1) Significant increase in knowledge (p=0.001), the proportion of HWs passing was 99.7% (HBB), 99.6% (ECEB) and 98.7% (IP). (2) 90.8% of HW achieved the passing score of 7/7 (100%) for the bag and mask skills test, 99.9% of HW passed the HBB OSCE (obtained at least a score of 16 out of 23), 99.9% of HW passed the ECEB OSCE (obtained at least a score of 20 out of 28). | The systematic approach to training, facilitation, improved training algorithms, strengthening the health system, and improving the availability of necessary equipment ensured sustainability. No long-term retention evaluation. No comparator group (pre-intervention comparison only). |
| Data et al. (2022) | Uganda  (Low-income) | To assess the effect of a peer learning intervention paired with mentorship on retention of HBB and ECEB skills, knowledge, and teamwork | Cluster-randomised trial | 125 healthcare workers (HCWs): primarily midwives (79.2%), clinical officers, and nurses  (Health facilities) | HBB and ECEB training | Laerdal Foundation for Acute Medicine; Elma Foundation; International Development Research Center (Canada) | Intervention: HBB/ECEB + peer learning + mentorship. Simulation-based training using NeoNatalie manikin, Peer learning cards with structured scenarios, Low-fidelity simulation, and WhatsApp communication for virtual support. Monthly peer practice for 12 months, mentorship visits at 2-3 months and 6-7 months post-training. Control: HBB/ECEB only. | January 2018 – February 2019 | 12 months post-training | (1) Skills retention, (2) Knowledge retention, (3) Teamwork skills | (1) Significant improvement in skills retention with peer learning intervention: HBB skills at 12 months (Intervention 57.9% vs Control 48.5% (p=0.007)) and ECEB skills at 12 months: (Intervention 61.7% vs Control 49.9% (p=0.004)). (2) No significant difference in knowledge decay over 12 months in both groups (Intervention 84% vs Control 82.9%, p=0.24). (3) No significant difference in teamwork skills between groups at 12 months (Intervention 70.7% vs Control 67.9%, p=0.19) | Structured mentorship visits, combined with additional follow-up without mentorship, provide a meaningful evaluation of the peer learning intervention’s sustained effect. Comparator: control arm receiving standard HBB and ECEB training only (no peer learning course or mentorship). |
| Dhungana et al. (2024) | Nepal  (Lower-middle-income) | To report overall changes in newborn outcomes in relation to a hybrid implementation approach for scaling up neonatal resuscitation training and skill retention | Prospective pre-post cohort study | 557 Physicians, Nurses, and Midwives  (Hospitals) | Helping Babies Breathe (HBB) training through the Safa Sunaulo Nepal (SSN) programme | Latter-day Saint Charities (LDSC) | Two-day HBB Training-of-Trainers (TOT) course; facility-based cascade training by trained facility-based trainers; low-dose high-frequency (LDHF) practice sessions; on-site mentoring by a single external mentor; monthly supervision, review meetings, refresher trainings, and monitoring of facility-based indicators. Hybrid approach during COVID-19: facility-based trainers provided in-person training while the mentor provided remote support via phone and Zoom, including virtual debriefing and review meetings. | January 2020 – February 2021 (14 months) | 14 months; baseline (first 3 months) vs endline (last 3 months) comparison | (1) Perinatal mortality (intrapartum stillbirths and neonatal deaths within 24 hours of life), (2) Neonatal deaths after 24 hours of life, (3) Newborn morbidity (sick newborn transfers) | Data collected from 18,712 deliveries over 14 months. (1) Significant decrease in intrapartum stillbirths by 23% (p=0.001) and neonatal deaths within the first 24 hours of life by 27% (p=0.005). (2) Significant decrease in neonatal deaths after first 24 hours of life by 41.3% (p<0.001). (3) No significant decrease in newborn morbidity (p=0.781). | COVID-19 (March 2020) forced a transition from in-person to hybrid (on-site + remote) support. Pre-COVID: on-site mentoring. During COVID: facility-based trainers provided in-person training while mentors provided remote support via phone/Zoom. Poor internet connectivity and trainee unfamiliarity with digital tools hampered programme efficiency. No comparator group (pre-intervention comparison only). |
| Ding et al. (2021) | Tanzania  (Lower-middle-income) | To evaluate a neonatal resuscitation training programme delivered over a two-year period for healthcare professionals in Zanzibar, Tanzania | Pre-post intervention study | 23 Physicians, Midwives, Nurses from 3 health sectors  (Hospital and health centers) | Neonatal Resuscitation Training (NRT) based on the American Heart Association (AHA) guidelines | Hunan Children’s Hospital (China); China Hunan Provincial Science and Technology Department | A 2-day training (theory classroom, hands-on simulation in a small group ratio of 1:5, handout, checklist) in Sept 2017, and nine months later, 2 days of refresher training were repeated in June 2018. | September 2017 – June 2018 | 9 months | (1) Knowledge, (2) Skills | (1) Significant increase in knowledge: in 2017, scores increased from 9.60 to 13.60 (95% CI: −5.900; −2.099, p<0.001); in 2018, scores increased from 10.80 to 15.44 (95% CI: −6.062; −3.217, p<0.001). Mean knowledge scores post-training over time improved from 13.60 in 2017 to 15.44 in 2018 (95% CI: −3.489; 0.190, p=0.030). (2) Significant increase in skills performance between the two time periods, from a mean of 32.26 (SD=2.35) to 42.43 (SD=1.73) (95% CI: −11.402; −8.945, p<0.001). | Language barrier with external trainers limited communication. Local practical context, shorter interval refresher training and ongoing support for local healthcare professionals are needed for programme sustainability. No comparator group (pre-intervention comparison only). |
| Ersdal et al. (2023) | Tanzania (Lower-middle-income) | To describe pre-implementation baseline data and halfway post-implementation trends in (1) adoption of clinical tools, (2) key clinical performance indicators, and (3) perinatal deaths (24-hour newborn mortality, fresh stillbirths [FSB], and maternal mortality) | Stepped-wedge cluster randomised implementation study | Healthcare workers at 30 facilities across 5 regions (Manyara, Tabora, Geita, Shinyanga, and Mwanza)  (Hospitals) | Safer Births Bundle of Care (SBBC), including Helping Mothers and Babies Survive programmes | Haydom Lutheran Hospital; Ministries of Health, Tanzania; SAFER (Stavanger University Hospital); Laerdal Global Health; UNICEF Tanzania | Four-component bundle: (1) innovative clinical tools (Moyo Fetal Heart Rate Monitor, NeoBeat Newborn Heart Rate Meter, Upright Newborn Bag and Mask); (2) innovative training tools (NeoNatalie Live Newborn Ventilation Trainer, MamaNatalie Postpartum Haemorrhage Simulator); (3) systematic continuous quality improvement (CQI) interventions driven by 2–3 trained facility champions per site, with weekly data feedback loops; (4) infrastructure and competence for sustainability. Low-dose high-frequency on-the-job simulation training guided by Helping Mothers and Babies Survive programmes. | March 2021 – December 2023 (halfway evaluation reporting data through July 2022) | Halfway evaluation: 5–13 months post-implementation depending on region | (1) Adoption of Moyo and NeoBeat devices, (2) Key clinical performance indicators, (3) 24-hour newborn mortality, (4) Fresh stillbirths, (5) Maternal mortality | Data from 138,357 deliveries (67,690 pre- and 70,667 post-implementation). (1) Moyo uptake ranged from 20–66% of deliveries; NeoBeat uptake 2.9–9.6% of newborns across regions. (2) Steady trends of decreased 24-hour newborn mortality in 4 of 5 regions; in Manyara (13 months, n=15,658), an estimated additional 100 newborns and 20 women saved. (3) FSB rates stable in 2 regions and increased in 3 regions post-implementation. (4) Steady trends of decreased maternal mortality in 4 of 5 regions. | Halfway evaluation; uptake of bundle varied between regions. Suspected underreporting of adverse outcomes at baseline. Enhanced focus on CQI component needed. No comparator group (stepped-wedge design with pre-intervention comparison). |
| Ezenwa et al. (2022) | Nigeria and Kenya  (Lower-middle-income) | To explore the feasibility and educational efficacy of using mobile virtual reality (VR) for precourse preparation of healthcare professionals in neonatal resuscitation training, comparing mobile VR simulation plus digitised HBB Provider's Guide versus digitised HBB Provider's Guide alone. | Secondary analysis of a randomised controlled trial | 179 nurses and nurse-midwives at 20 secondary and tertiary healthcare facilities  (Hospitals) | HBB training | University of Washington; Indiana University; University of Lagos; Alupe University College, Kenya; University of Oxford | **VR group:** Mobile VR headset + study phones with eHBB app + digitised guide. **Control group**: Digitised HBB Provider's Guide only. Equipment: Low-cost VR headset, Android phones, Neonatalie Advanced manikin. Follow-up: Standard in-person HBB training for both groups after assessments | December 2018 – February 2020 | Immediately post-training only | (1) Knowledge, (2) Skills | Only 3.4% (6/178) of all participants achieved passing scores. Similar overall scores between groups on all three assessments (p>.05). (1) No significant difference in knowledge between groups (VR group median: 15/18 (IQR 14-16) and Control group median: 16/18 (IQR 15-17), p=0.29). (2) Significant difference in skills with VR group performed better: head positioning and airway clearing ( 86% vs. 65% (p=.002)), Removing wet cloth: (38% vs. 25% (p=0.08, trending)), Head tilt during ventilation: 75% vs. 62% (not significant, p=.35)), with control group performed better: Identifying helper/emergency plan: 18% vs. 8% (p=.045) and Hand washing: 36% vs. 22% (p=.048) | The widespread ownership of smartphones in low-resource settings enabled the feasible use of accessible mobile VR simulations for pre-course training in neonatal resuscitation. |
| Farhadi et al. (2023) | Iran  (Upper-middle-income) | To evaluate the impact of team-based simulation training on the development of neonatal resuscitation competency in senior paediatric residents' neonatal resuscitation competency | Intervention educational study | 21 senior paediatric residents (second and third year) at Boo Ali Sina Hospital, Sari  (Hospital) | Neonatal Resuscitation Program (NRP), 8th edition (2021) | National Agency for Strategic Research in Medical Education, Tehran, Iran | Online preparatory class on NRP 2021 updates, followed by in-person workshop (manikins, multimedia, theoretical methods). Individual NRP advanced megacode assessment. Team-based simulation in 3-person groups using HBB manikin (Laerdal Medical) in simulated NICU environment. Two resuscitation scenarios (preterm and term) with video-recorded performances and structured plus-delta debriefing. Kirkpatrick model levels 1–3 used for evaluation. | February 2022 – May 2022 | 4 months (OSCE assessment for knowledge retention) | (1) Knowledge and confidence (pre/post MCQ, 20 items), (2) Skills (NRP megacode assessment; OSCE at 4 months), (3) Satisfaction (20-item survey checklist) | (1) Significant increase in overall knowledge score (pre-test 62.6 ± 13.7 to post-test 86.3 ± 9.5, p=0.001); largest gains in chest compression and team briefing. (2) Mean megacode score 35.6 ± 2.2 (out of 38); 18/21 residents passed on first attempt. (3) At 4 months, all residents passed OSCE (mean 8.6 ± 1.1 out of 10), demonstrating retention. (4) Overall satisfaction score 96.3 ± 3.7 (out of 100). | First neonatal resuscitation team-based simulation study in Iran. Low-cost local materials (HBB manikin) used in resource-limited setting. No randomised control group. Small sample size (n=21). |
| Fritz et al. (2020) | Mexico  (Upper-middle-income) | To evaluate the effect of PRONTO on knowledge and self-efficacy on obstetric and neonatal emergency care among health providers, and assess heterogeneities by gender, age, shift, and profession | Before-after evaluation design | 351 healthcare providers (obstetrician-gynaecologists, paediatricians, general practitioners, interns, nurses, and midwives) across 14 hospitals in 6 states  (Hospitals) | PRONTO (Neonatal and Obstetric Rescue Program: Optimal and Timely Treatment) | National Institute of Public Health of Mexico (INSP); National Institute of Women (INMUJERES); University of Washington; University of California San Francisco | Two-module interprofessional training: Module I (16 hours over 2 days) on obstetric haemorrhage, neonatal resuscitation, teamwork, and communication; Module II (8 hours, 3 months later) on preeclampsia/eclampsia and shoulder dystocia. In-situ simulations using PartoPants™ hybrid birth simulator and NeoNatalie© manikin in labour/delivery rooms. Video-guided debriefings, interactive team-building exercises, and targeted skills sessions. | June 2013 – January 2014 | Immediately post-training only | (1) Knowledge, (2) Self-efficacy | (1) Significant knowledge gain by an average of 19 percentage points for haemorrhage, 23 for neonatal resuscitation, 19 for shoulder dystocia, and 15 for preeclampsia/eclampsia (p<0.001). (2) Self-efficacy increased significantly by 16 percentage points on average. | No long-term follow-up or health outcomes measured. Night shift workers showed lower knowledge scores. Male doctors more likely to drop out. Nurses gained most knowledge but started with lowest scores. No comparator group (pre-intervention comparison only). |
| Gamtessa et al. (2020) | Ethiopia  (Low-income) | To assess the effectiveness of Helping Babies Breathe (HBB) and Essential Care for Every Baby (ECEB) training on the satisfaction and knowledge of trainees in SNNPR, Ethiopia, using a Kirkpatrick training evaluation model | Formative evaluation (Kirkpatrick training evaluation model, levels 1 and 2) | 98 healthcare providers: nurses (30.6%), midwives (56.1%), health officers (9.2%), and anaesthetists (4.1%)  (Hospitals) | HBB and ECEB training | Village Health Partnership | Four-day training in 5 sub-groups (trainer-to-trainee ratio 1:5). Six trained senior lecturers (3 paediatric nursing specialists, 2 general practitioners, 1 clinical midwife) facilitated using HBB and ECEB action plans, provider guides, facilitator guides, flipcharts, and materials for demonstration. Five training rounds conducted across two hospitals. | 2018 – 2019 (five training rounds) | Immediately post-training only | (1) Knowledge, (2) Satisfaction | (1) Significant increase in HBB knowledge score from 64.42 (SD ± 17.43) to 80.71 (SD ± 14.36) (t(97)=11.146, p<0.001). Significant increase in ECEB knowledge score from 59.10 (SD ± 13.18) to 73.73 (SD ± 14.17) (t(97)=11.684, p<0.001). (2) Mean satisfaction score was 32.88 (SD ± 2.68); 93.88% of trainees scored ≥ mean. Updated parts of training (suction, ventilation, cord care, skin-to-skin) rated most useful. | Evaluation limited to Kirkpatrick levels 1 (satisfaction) and 2 (knowledge) only. No long-term retention, skills assessment, or clinical outcomes evaluated. Small sample size. |
| Hatamleh et al. (2021) | Jordan  (Lower-middle-income) | To evaluate the effectiveness of the HBB training programme on midwives’ knowledge and skills in Northern Jordan | Quasi-experimental nonequivalent control group design | 50 midwives (control, n=25; experimental, n=25) from 3 public hospitals in Northern Jordan  (Hospitals) | HBB training | Not reported | Experimental group: 2-hour HBB teaching session, 1-hour knowledge testing, and 25-minute individual skills assessment using flip chart, pocket-sized workbook, NeoNatalie mannequin, and bag-mask with scripted scenario. Control group: no HBB training; tested on knowledge and skills only. | August 2016 – November 2016 | 8 months | (1) Knowledge, (2) Skills | 8-month post-training: (1) Significant difference in knowledge between groups (mean score experimental vs control: 16.18, SD=0.85 vs 14.24, SD=3.44, p=0.000), (2) Significant difference in all skills (mean score experimental vs control: OSCE A 10, SD=1.02 vs 6.2, SD=1.58, OSCE B 14.68, SD=1.64 vs 5.67, SD=1.78, Bag-Mask Ventilation 5.13, SD=0.99 vs 2.12, SD=1.01, p=0.000, all) | First HBB study in Jordan. Limited to public hospitals in Northern Jordan; may not be generalisable. Quasi-experimental design may limit causal inference. No data on sustained outcomes between immediate post-intervention and 8-month follow-up. |
| Heard Stittum et al. (2023) | Ethiopia  (Low-income) | To assess whether standard HBB training supplemented with an educational clinical video on newborn resuscitation improves knowledge and skills retention compared to standard HBB training alone | Cluster-randomised controlled trial | 84 midwives from 2 public teaching hospitals (Tikur Anbessa Hospital and St. Paul’s Hospital Millennium Medical College, Addis Ababa)  (Hospitals) | Helping Babies Breathe (HBB), supplemented with Global Health Media Project (GHMP) educational clinical video | Laerdal Foundation; Global Health Media Project (GHMP); Vermont Oxford Network | Cluster-randomised into 14 clusters (7 per arm) of 6 participants. Control: standard full-day interactive HBB provider course (average 5 trainees per trainer). Intervention: standard HBB training plus 15-minute GHMP educational clinical video on newborn resuscitation (viewed pre-training, with ongoing access via digital messaging application). Follow-up assessments at 1, 3, and 7 months by HBB master trainers. | November 2022 – June 2023 | 1, 3, and 7 months post-training | (1) Knowledge (HBB 18-item knowledge check), (2) Skills (bag-and-mask skill check, OSCE A, OSCE B) | (1) No significant difference in knowledge pass rates between groups at any time point; >97% of both groups passed at 7 months (p=1.0). (2) No significant difference in bag-and-mask skill pass rates at 7 months (control 51.2% vs intervention 41.9%, p=0.39). (3) No significant difference in OSCE A at 7 months (control 73.2% vs intervention 72.1%, p=0.92) or OSCE B (control 75.6% vs intervention 79.1%, p=0.71). Addition of video did not influence skill retention. Midwives did not show expected knowledge and skill loss over time. | 100% follow-up at 7 months. Frequent reassessments by HBB master trainers may have contributed to absence of expected knowledge/skill decline. Only 40.5% of intervention group reported referencing a video. Participants from two tertiary academic centres; may not be generalisable. |
| Higgins et al. (2020) | India  (Lower-middle-income) | To assess facility-level neonatal resuscitation (NR) skill retention after PRONTO simulation training within the AMANAT mentoring programme | Prospective observational study | Auxiliary nurse midwives/ANMs, General nurse midwives/GNM across 64 facilities.  (Primary health centres) | PRONTO International simulation training integrated within the AMANAT mentoring program | PRONTO international | In situ, intrapartum simulation training including teamwork and communication exercises, skill stations, and case-based learning, focusing on mother–infant dyad care. Initial 8-month training phases facilitated by college-educated nurse mentors (BSc/MSc) with monthly NR simulations. Repeat training (AMANAT Jyoti) facilitated by peer ANM/GNM mentors with single simulation sessions. Assessment simulations video-recorded for debriefing. | September 2015 – February 2019 | 15–29 months (training gap between AMANAT post-assessments and AMANAT Jyoti baseline reassessments) | (1) Skills acquisition (facility-level NR skill change from mid to post initial training), (2) Skills retention (facility-level NR skill change over training gap), (3) Skills reacquisition with peer-facilitated refresher training | Data from 192 assessment simulations at 64 primary health centres (PHCs). (1) Significant improvement from mid to post initial training in PPV with chest rise (31.1 percentage point increase, p=0.03) and heart rate assessment (13.1 percentage point increase, p=0.03). (2) Significant skill deterioration over the 15–29 month training gap: PPV with chest rise declined by 26.2 percentage points (p=0.01); heart rate assessment declined by 20.9 percentage points (p<0.01). No significant changes in stimulation, suction, neck extension, or rate of PPV. (3) With peer-facilitated refresher training (n=45 PHCs, 90 simulations), significant improvement in heart rate assessment (16.1 percentage point increase, p=0.04); no significant change in other skills assessed. | Due to high staff turnover, not all ANMs/GNMs in AMANAT Jyoti had received prior PRONTO training; exact percentage unknown. Facility-level assessment risks overestimating skill decline if untrained nurses entered previously trained PHCs. Supply issues (appropriately sized masks, watches for timekeeping) identified as additional barriers to PPV and heart rate assessment. No comparator group (pre-intervention comparison only). |
| Innerdal et al. (2021) | Mali  (Low-income) | To evaluate the effect of Helping Babies Breathe (HBB) training on perinatal mortality rate at a district hospital in Kati district, Mali | Single-arm prospective intervention study | 305 birth attendants (including 16 midwives, 12 obstetrical nurses, 1 gynaecologist at the district hospital; additional birth attendants across 33 community health centres)  (First-level referral hospital and community health centres) | HBB training | NorSahel (Norwegian NGO); Koulikoro Health Region, Mali; Oslo University Hospital, Norway | HBB 1st edition training (1–2 day sessions) using train-the-trainer principle: 5 master trainers trained, then 45 facilitators trained across district. All birth attendants trained on-site at the district hospital and 33 community health centres. Facilities equipped with bag and mask and resuscitation training materials (none available prior to intervention). HBB 2nd edition introduced in 2017 with weekly repetition training and re-training of all facilitators over 3-day courses. | April 2016 – December 2018 (pre-HBB period: January 2015 – March 2016) | Comparison of pre-HBB (January 2015 – March 2016) and post-HBB (April 2016 – December 2018) periods; additional comparison between HBB 1st and 2nd edition periods | (1) Perinatal mortality rate (PMR: fresh stillbirths plus neonatal deaths within first 24 hours per 1,000 births), (2) Fresh stillbirth rate (FSR), (3) Very early neonatal mortality rate within 24 hours (VENMR 24h), (4) Resuscitation rate | Data from 9,769 births (3,125 pre-HBB; 6,644 post-HBB). (1) Significant reduction in PMR from 21.7/1,000 to 6.0/1,000 (RR 0.27, 95% CI 0.19–0.41, p<0.0001). (2) Significant reduction in FSR from 15.7/1,000 to 5.3/1,000 (RR 0.33, 95% CI 0.22–0.52, p<0.0001). (3) Significant reduction in VENMR (24h) from 6.3/1,000 to 0.8/1,000 (RR 0.12, 95% CI 0.05–0.33, p<0.0001). (4) No further significant reduction after introducing HBB 2nd edition (PMR 6.1 to 5.9/1,000, RR 0.96, 95% CI 0.53–1.83, p=0.97). | Local ownership drove the programme’s success; the district health chief was a co-author and made all final implementation decisions. Facilities had no bag and mask equipment before the intervention. Security situation in Mali (including terror attacks against western groups) was a major obstacle, forcing the project group to often meet in Dakar, Senegal. First study to evaluate HBB in a French-speaking country and in western Africa. No comparator group (pre-intervention comparison only). |
| Kankindi et al. (2020) | Rwanda  (Low-income) | To assess HBB knowledge and skills retention at two days and six weeks post-training among midwives and nurses working in selected health centres in Kigali, Rwanda | Quasi-experimental pre-post study | 60 midwives and nurses (90% nurses, 10% midwives) from 4 health centres  (Health centres) | HBB training | Not reported | One-time two-day HBB training session (2nd edition) at each work site, including lectures, slide presentations, interactive Q&A sessions, group discussions, and hands-on exercises using NeoNatalie mannequin. Knowledge assessed by 18-item MCQ at pre-training, two days post-training, and six weeks post-training. Skills assessed by OSCE (24-item checklist) at two days and six weeks post-training. | March 20 – May 8, 2019 (approximately 7 weeks) | 6 weeks | (1) Knowledge retention (18-item MCQ at pre-test, 2 days, and 6 weeks post-training), (2) Skills retention (24-item OSCE at 2 days and 6 weeks post-training) | (1) Knowledge overall mean scores improved significantly from pre-test (8.75, SD=3.98) to 2 days post-training (16.90, SD=1.29, p<0.01); slight decrease at 6 weeks (16.27, SD=1.50) but still significantly higher than pre-test (p<0.01). (2) Skills mean scores decreased significantly from 2 days post-training (21.77, SD=1.45) to 6 weeks (19.73, SD=2.21, p<0.01). Previous HBB training was the only sociodemographic characteristic significantly associated with knowledge performance at 2 days and 6 weeks (p<0.05). | No OSCE was done in the pre-training period. Same MCQ and OSCE were used at all time points, potentially allowing self-study improvement. Majority of participants (78.3%) had not attended any previous HBB training. Findings cannot be generalised as the study was conducted in only one district with four health centres. No comparator group (pre-intervention comparison only). |
| Lima et al. (2023) | Brazil  (Upper-middle-income) | To evaluate neonatal outcomes after the implementation of the Neonatal Resuscitation Program of the Brazilian Society of Pediatrics (PRN-SBP) in a region of high socioeconomic vulnerability in Brazil | Pre-post intervention study | 431 healthcare professionals (65 of 87 doctors [74.7%], including 10 of 11 paediatricians and 14 of 17 obstetricians; plus nurses, physical therapists, nursing technicians, and nursing assistants) across 5 secondary healthcare regions supporting 62 cities  (Hospitals) | The Neonatal Resuscitation Program of the Brazilian Society of Paediatrics | Piaí State Department of Health (SESAPI); Brazilian Society of Pediatrics (SBP) | PRN-SBP courses (8 hours each) comprising two theoretical and four practical lessons, taught exclusively by a PRN-SBP-accredited paediatrician instructor. Three course types: resuscitation of ≥34-week newborns, resuscitation of <34-week newborns, and transport of high-risk newborns. Each course trained 6–8 participants. Pre- and post-test with 25 MCQs (80% passing score). 700 training sessions conducted across 106 courses. Delivery room structuring with acquisition of essential resuscitation equipment. Fortnightly follow-up via social networks for 12 months. | March 2018 – June 2018 (training); follow-up assessment June–July 2019 | Immediately post-training and 12 months after intervention (knowledge assessed in sample of 167 healthcare professionals) | (1) Delivery room structuring (percentage of materials required for neonatal resuscitation), (2) Knowledge retention (25-item MCQ, 80% passing score), (3) Neonatal outcomes (delivery room mortality, number of newborns resuscitated, NICU transfers) | (1) Average materials availability increased from 28.4% pre-intervention to 80.6% immediately post-intervention and 83.0% at 12 months; all five sites achieved 100% of fundamental items. (2) Post-training approval rate of 95.5%; satisfactory knowledge retention at 12 months with minimal score difference between periods. (3) 72.6% reduction in delivery room mortality across the five study sites; 479 newborns resuscitated (86.4% ≥34 weeks); 459 registered as survivors (95.8%). Significant increase in NICU transfers (100% increase at reference NICU). | Conducted in the southwestern mesoregion of Piauí, a remote region with high socioeconomic vulnerability, high underreporting of neonatal mortality, and a health system weakened by the lack of paediatricians and obstetricians (only 11 paediatricians for five health regions). Prior to intervention, 80% of sites lacked auto-inflatable balloons, making positive airway pressure impossible. No pre-intervention resuscitation data available for comparison. No comparator group (pre-intervention comparison only). |
| Mayer et al. (2022) | South Africa  (Upper-middle-income) | To determine and compare the fresh stillbirth rate (FSBR), early neonatal mortality rate (ENMR), and neonatal mortality rate (NMR) between 2015 (baseline) and subsequent years up to 2020 following implementation of HBB training in five hospitals in the Oliver Reginald Tambo (ORT) District, South Africa | Retrospective observational study | 4,795 healthcare providers trained over 5 years (16.7% as facilitators); including midwives, nurses, and doctors across 2 district hospitals, 2 regional hospitals, and 1 tertiary hospital  (2 district hospitals, 2 regional hospitals, and 1 tertiary hospital) | HBB training | American Academy of Pediatrics (AAP) through Ronald McDonald House Charities; Resuscitation Council of Southern Africa | HBB training using train-the-trainer model. Full-day annual HBB training covering all scenarios conducted at all hospitals. Hospitals C, D, and E had full-time on-site midwife mentors with advanced midwifery training. Hospitals A and B visited by mentors from hospitals C and E every 3 months or when needed. Trained healthcare providers encouraged to continue training others at their own hospitals. | September 2015 – December 2020 (5 years; 2015 as baseline pre-HBB period) | During implementation period only | (1) Fresh stillbirth rate (FSBR: neonates ≥500g born with no signs of life and no maceration per 1,000 total births), (2) Early neonatal mortality rate (ENMR: deaths within first 7 days per 1,000 live births), (3) Neonatal mortality rate (NMR: deaths within first 28 days per 1,000 live births) | Data from 123,898 total births across five hospitals. (1) No significant change in FSBR (OR 0.93, 95% CI 0.79–1.09, p=0.363); no significant trend over time (r²=0.0, p=0.984). (2) Significant reduction in ENMR (OR 0.78, 95% CI 0.70–0.87, p<0.001); significant linear trend reduction over time (r²=0.45, p=0.001); ENMR decreased from approximately 20 to under 12 per 1,000 live births. (3) Significant reduction in NMR (OR 0.81, 95% CI 0.73–0.90, p<0.001); significant trend over time (r²=0.23, p=0.026). Individual hospital analysis showed significant ENMR reduction only in Hospital A (r²=0.61, p<0.001) and Hospital E (r²=0.19, p=0.048); no significant changes in regional hospitals. | Variations in outcomes across hospitals suggest other factors beyond HBB training affected perinatal/neonatal outcomes. High attrition rate of trainers and trained staff due to frequent movement and relocation in the province. In district hospitals, junior doctors with no neonatal care experience cover all hospital sections, leading to skill degeneration and compromised continuity of care. Rural setting with vast geographical distances between hospitals challenged follow-up mentoring. Authors recommend telesimulation and digital platforms for future training. No comparator group (pre-intervention comparison only). |
| Mubeen et al. (2021) | Pakistan  (Lower-middle-income) | To evaluate the effectiveness of simulation-based high-frequency recurring training of Helping Babies Breathe (HBB) for Community Midwives (CMWs) in district Gujrat, Pakistan | Pre-post intervention study | 50 Community Midwives (CMWs) recruited (43 enrolled in first training; 34 completed all three sessions)  (Community birthing centres) | Helping Babies Breathe (HBB) 2nd edition | International Confederation of Midwives (ICM) through Marrie Goubran Award; Midwifery Association of Pakistan (MAP); IRMNCH Programme, Punjab | Simulation-based high-frequency recurring training. One-day training of trainers (3 CMW tutors plus 3 research team members). Three one-day training sessions for CMWs at 2-month intervals (September 2017, November 2017, January 2018): initial 8-hour workshop followed by two refresher sessions of 6 and 5 hours. Each session included HBB content delivery via presentations and flip charts, skills demonstration in large group, practice in groups of 5–6 with one facilitator, bag-mask ventilation sign-off, and pre/post knowledge and skills assessments. Between sessions, trained supervisors randomly visited 10% of participants monthly with NeoNatalie simulator for on-site skill assessment and monitoring. | May 2017 – March 2018 (11 months total; training September 2017 – January 2018) | During implementation period only | (1) Knowledge (18-item MCQ; 80% pass), (2) Skills (OSCE A [routine care/Golden Minute, 12 items, pass ≥9] and OSCE B [OSCE A + bag-mask ventilation + heart rate decision-making, 23 items, pass ≥17]), (3) Participant satisfaction with training, (4) Newborn morbidity and mortality indicators (deliveries, live births, asphyxia, referrals, stillbirths) | Data from 34 CMWs who completed all three sessions. (1) Significant improvement in knowledge after training 1 (50% to 97.1% pass, p<0.0001) and training 2 (64.7% to 88.2% pass, p=0.008); no significant change after training 3 (91.2% to 97.1%, p=0.625), indicating retention. (2) Significant improvement in OSCE A after training 1 (8.8% to 85.3% pass, p<0.0001) and training 2 (70.5% to 100%, p=0.002); no significant change after training 3 (91% to 100%, p=0.25). (3) Significant improvement in OSCE B after all three trainings (p<0.05). (4) Decreasing trend in stillbirth rates (20.5 to 19.8 at midline and 19.6/1,000 at endline); increased asphyxia identification (3.6% to 6.6%). Significant differences in morbidity and mortality outcomes could not be detected due to small sample size and short intervention period. | Lowest scores observed in baseline pre-test, indicating deployed CMWs were not well-versed in evidence-based newborn resuscitation approaches. CMWs work as private practitioners at distant locations, making on-site low-dose training with weekly coaching not feasible. Training facilitated by utilising existing local community midwifery tutors (no master trainers hired), ensuring sustainability. Study conducted in one district with small sample; findings cannot be generalised. Community-based MIS largely depended on CMWs’ self-reported data, limiting mortality outcome assessment. No comparator group (pre-intervention comparison only). |
| Munir et al. (2021) | Pakistan  (Lower-middle-income) | To assess the improvement in knowledge of healthcare professionals after attending the Neonatal Life Support (NeoLiS) training workshop | Pre-post intervention study | 1,350 participants across 52 workshops: 1,080 (81%) doctors (consultants and post-graduate residents), 257 (18%) nurses, and 13 (1%) paramedics and rescue workers  (Hospitals) | Neonatal Life Support (NeoLiS) training, based on ILCOR guidelines and endorsed by the Pakistan Paediatric Association (PPA) | Neonatal Resuscitation Council of Pakistan Paediatric Association (PPA); Directorate of NeoLiS | Hands-on workshops (25–30 participants each) based on ILCOR guidelines. First part influenced by HBB (Golden Minute technique), continuing to drug administration. Workshops regularly arranged and monitored by Neonatal Resuscitation Council of PPA. Simpler curriculum adaptable to facility level: initial part (up to airway management) taught to nurses and midwives at level-I facilities; full algorithm including drug injection taught to doctors and nurses at level-III hospitals. Knowledge assessed by 15-item true/false questionnaire pre- and post-workshop. | September 2017 – June 2018 | Immediately post-workshop only (no long-term follow-up) | Knowledge (15-item true/false questionnaire on resuscitation skills, assessed pre- and post-workshop) | Significant improvement in mean overall knowledge score from 71.96% (SD=8.38) pre-workshop to 86.60% (SD=5.48) post-workshop (p<0.001). Significant improvement across all professional categories: doctors (73.41% to 87.99%, p<0.01), nurses (66.84% to 82.74%, p<0.01), and paramedics (52.26% to 75.80%, p<0.01). Overall scores of nurses and paramedics were lower than doctors, likely due to differences in background knowledge. | National programme endorsed by Pakistan Paediatric Association, with 52 workshops conducted across 2 years (35 in 2017, 17 in 2018). Workshop designed to be adaptable: initial part (up to airway management) for level-I facilities; full algorithm for level-III hospitals. Only immediate post-workshop knowledge assessed; no long-term retention or clinical outcomes evaluated. No comparator group (pre-intervention comparison only). |
| Munyaw et al. (2022) | Tanzania  (Lower-middle-income) | To assess the impact of Helping Babies Breathe (HBB) training coupled with Moyo Doppler fetal heart rate (FHR) monitoring on healthcare workers’ skills and perinatal mortality | Quasi-experimental pre-post study | 107 health care workers (29 doctors, 78 nurses/midwives) at 3 district hospitals (Mbulu, Babati, Hanang)  (Hospitals) | Helping Babies Breathe (HBB) programme coupled with Moyo Doppler fetal heart rate (FHR) monitoring device | Laerdal Foundation; Haydom Lutheran Hospital, Tanzania | HBB training with Moyo Fetal Heart Rate (FHR) Monitor coupled with neonatal resuscitation simulation training (master trainers conducted a trainer of trainees, hands-on, simulator approach). Hospitals received equipment (Moyo devices, simulators, and bag-mask ventilators). | 2018 – 2019 (2 years) | 2 years (2018–2019, compared with 2017 baseline) | (1) Knowledge (knowledge check questions pre- and post-training), (2) Skills (OSCE A and OSCE B pre- and post-training), (3) Perinatal outcomes: fresh stillbirths (FSB), early neonatal deaths within 24 hours, and early neonatal deaths within 7 days (2017 baseline vs 2018–2019 implementation) | (1) Significant increase in knowledge post-training by 13.6% (p<0.001) and skills by 25.5% (OSCE A) and 38.2% (OSCE B) (p<0.001). (2) No significant reduction in fresh stillbirths (p=0.506); significant reduction in early neonatal deaths within 24 hours (p=0.036) and within 7 days (p=0.036). | First scale-up project to roll out the Moyo FHR monitor coupled with HBB training at lower-level health facilities in Tanzania. Local Trainers of Trainees (TOTs) supported ongoing practices to sustain knowledge and skills. Previous studies using Moyo alone did not show perinatal mortality reduction without accompanying resuscitation training. Some trained health care workers were shifted to other departments or retired during implementation. No comparator group (pre-intervention comparison only). |
| Neupane et al. (2022) | India  (Lower-middle-income) | To assess whether simulation-based education (SBE) improves practices and knowledge of junior residents for stabilisation of preterm neonates in the delivery room compared to conventional education (CE) | Open-label randomised controlled trial | 24 first- and second-year paediatric residents (12 SBE, 12 CE)  (Hospital) | Custom-designed curriculum for initial stabilisation of preterm neonates (physiology, equipment preparation, thermal care, and respiratory support) | All India Institute of Medical Sciences (AIIMS), New Delhi, India | Simulation-Based Education (SBE): one-time session including orientation (equipment pre-brief), actual scenario run, and debriefing; used moderate-fidelity manikin (Premature Anne with SimPad Plus, Laerdal Medical). Conventional Education (CE): self-demonstrations on task trainers (NeoNatalie newborn simulator, Laerdal Medical) with facilitator feedback. Equal duration and facilitator-participant ratio in both arms. | January 2018 – July 2019 (single-day workshop per session) | Within 8 weeks post-training (real-time performance evaluation on preterm neonates) | (1) Skills (OSCE stations assessed same day post-training), (2) Knowledge (pre- and post-test written assessment), (3) Real-time Preterm Stabilisation Performance Evaluation (PSPE) score (video-recorded clinical performance on preterm neonates 31–36 weeks within 8 weeks post-training) | (1) No significant difference in skill scores between SBE and CE groups (mean difference 4.6; 95% CI −2.1 to 11.3, p=0.17). (2) No significant difference in knowledge gain between groups (mean difference 0.91; 95% CI −1.81 to 3.64, p=0.49). (3) No significant difference in real-time Preterm Stabilisation Performance Evaluation (PSPE) scores (mean difference −2.68; 95% CI −8.35 to 13.71, p=0.62). | A single session of SBE did not improve junior residents’ skills, real-time performance, or knowledge compared to conventional task training when controlled for confounding by duration of training. The eight-week follow-up was chosen because literature suggests reasonable skill deterioration thereafter. Cross-contamination between groups possible. Small sample size (n=24). |
| Nishimwe et al. (2021) | Rwanda  (Low-income) | To assess the effect of the Safe Delivery Application (SDA) mLearning intervention on nurses’ and midwives’ knowledge and skills for the management of postpartum haemorrhage (PPH) and neonatal resuscitation (NR) | Pre-post intervention study | 54 Nurses, Midwives at 2 hospitals.  (Hospital) | The Safe Delivery Application (SDA) is based on WHO guidelines on BEmONC | Maternity Foundation; Copenhagen University; University of Southern Denmark; Consortium for Advanced Research Training in Africa (CARTA) | Half-day SDA introduction training (description of SDA features and modules, how to use smartphone and SDA, joint video viewing, interactive exercises in small groups). Six-month personalised mLearning journey using the SDA on personal or researcher-provided smartphones to train knowledge and skills on managing obstetric and neonatal emergencies. Twice-weekly monitoring visits by researcher and research assistants for encouragement and guidance. | July 2019-April 2020 | 6 months post-SDA introduction | (1) Knowledge (self-administered key feature questionnaire on PPH management and NR), (2) Skills (self-administered questionnaire with simulated clinical cases for PPH and NR) | Significant increases in PPH knowledge (mean difference 17.1%; 95% CI 14.69–19.49, p<0.001, Cohen’s d=1.8) and PPH skills (mean difference 2.6%; 95% CI 1.01–4.25, p=0.002, Cohen’s d=0.3). Significant increases in NR knowledge (mean difference 19.1%; 95% CI 16.31–21.76, p<0.001, Cohen’s d=1.7) and NR skills (mean difference 5.5%; 95% CI 3.66–7.41, p<0.001, Cohen’s d=0.7). Improvements unaffected by in-service training or smartphone experience. | Challenges to implementation of quality BEmONC care include gaps in resources and the regulatory environment. Skills assessed via self-administered simulated clinical cases, not real practice. Small sample (n=54) from two hospitals limits generalisability. No comparator group (pre-intervention comparison only). |
| Nishimwe et al. (2022) | Rwanda  (Low-income) | To investigate the use of the Safe Delivery mHealth Application (SDA) and its relationship to basic emergency obstetric and newborn care (BEmONC) outcomes for the most frequent birth-related complications (postpartum haemorrhage and newborn asphyxia) in Rwanda | Pre–post intervention study | 54 nurses and midwives at 2 district hospitals (Masaka and Nyamata) (Hospitals) | The Safe Delivery Application (SDA) is based on WHO guidelines on BEmONC | Ministry of Health Rwanda, Maternity Foundation, University of the Witwatersrand | In-person half-day training session on SDA use, hands-on practice with SDA, use of personal smartphones and backup smartphones provided by researcher. Twice-weekly monitoring visits from research assistants. Six-month self-directed learning via SDA features (animated instruction videos, action cards with clinical guidelines, essential drugs lists, practical procedures guidelines, and MyLearning platform with quizzes at familiar, proficient, and expert levels). | October 2019 – March 2020 | 6 months | (1) Neonatal outcomes following neonatal resuscitation (stable [skin-to-skin], unstable [transfer to NICU], death), (2) Maternal outcomes following PPH management (stable, unstable [transfer to referral hospital], death) | (1) Significant improvement in neonatal outcomes: stable outcomes increased from 31% to 72%, transfers to NICU decreased from 62% to 28%, deaths decreased from 7% to 0% (p=0.000, Fisher’s exact test). (2) Significant improvement in maternal outcomes: stable outcomes increased from 78% to 94%, transfers decreased from 19% to 6%, deaths decreased from 3% to 0% (p=0.048, Fisher’s exact test). | mHealth interventions focusing on clinical decision support in maternal and newborn care may contribute to improved service delivery and should be considered by policymakers in resource-limited settings. Pre-post design limits causal attribution; outcomes may have been influenced by factors other than the SDA. No comparator group (pre-intervention comparison only). |
| Nvonako et al. (2022) | Cameroon  (Lower-middle-income) | To evaluate the effect of in-hospital training using simulations on the competence (knowledge and skills) of health care workers in newborn resuscitation | Quasi-experimental pre-post study | 30 Midwives, nurses, and paramedical staff  (Hospitals) | WHO Emergency Triage Assessment and Treatment (ETAT+) Course | University of Nairobi, Kenya; Pan African Medical Journal – Centre for Public Health Research and Information; Cameroon Baptist Convention Health Services | In-hospital training using WHO adapted ETAT+ guideline on newborn resuscitation. Three groups of 10 participants trained over one week (1.5 hours per day per group): 35-minute didactic lecture using ETAT+ PowerPoint presentation, 10 minutes of skills demonstration with mannequins using 2 case scenarios (meconium and no meconium), 45 minutes hands-on practice by all participants. Real-time observation of skills during routine deliveries before and after training. Three key informant interviews and one focused group discussion on barriers to effective resuscitation. | November – December 2019 (5 weeks) | Within 1 week post-training (knowledge re-assessment and real-time skills observation over 2 weeks) | (1) Knowledge (standardised NRP questionnaire; pass mark ≥70%), (2) Skills (real-time observation of newborn resuscitation using WHO ETAT+ checklist; 5 key steps required for pass), (3) Skills (simulation scenarios with mannequins using ETAT+ checklist) | (1) No significant difference in knowledge pass rates before and after training (53% in both; McNemar’s test p=1.00). (2) Significant increase in real-time skills scores from median 54% (IQR=14) to 82% (IQR=30) (p<0.0001). (3) Significant increase in simulation skills: scenario 1 (no meconium) from 43% to 57% (p=0.008); scenario 2 (meconium) from 38% to 75% (p<0.0001). | Barriers to effective newborn resuscitation identified: lack of appropriate equipment (bags and masks), inadequate knowledge in health workers, shortage of trained staff, and poor teamwork between midwives and anaesthetists in theatre. Study introduced WHO ETAT+ structured approach to the facility. Skills assessed both in real-time practice and simulation scenarios. Single-site study with sole observer (PI). No comparator group (pre-intervention comparison only). |
| Nyiringango et al. (2021) | Rwanda  (Low-income) | To assess the impact of a practice-based mentorship programme on nurses’ and midwives’ knowledge about and self-efficacy for neonatal resuscitation | Quasi-experimental pre-post study | 141 Nurses, Midwives from 68 health centres across 3 districts.  (Health centers) | Training, Support, and Access Model (TSAM) practice-based mentorship programme incorporating Helping Babies Breathe (HBB) educational programme | Western University, Canada; Global Affairs Canada (Government of Canada); Rwandan Ministry of Health | Practice-based one-to-one mentorship at health centre level. Mentors (midwives with MNCH expertise from district hospitals) received a two-week educational session on emergency obstetrics including neonatal resuscitation and mentoring skills. Mentors visited mentees monthly at their workplaces over 6 months (6 visits total), working together on delivery cases including neonatal resuscitation using HBB programme steps. Mentees observed mentor technique, then performed resuscitation with constructive feedback. Ongoing telephone support between visits. | 6 months (mentorship programme period) | Immediately post-mentorship programme (6 months after pre-assessment) | (1) Knowledge about neonatal resuscitation (18-item multiple-choice questionnaire adapted from HBB knowledge assessment tool), (2) Self-efficacy for neonatal resuscitation (24-item scale on 10-point Likert scale; Cronbach alpha=0.93) | (1) Significant increase in knowledge from pre- to post-mentorship (mean 78.6%, SD=12.2 to 93.38%, SD=8.4; mean difference=15.17, 95% CI 12.8–17.6). (2) Significant increase in self-efficacy from pre- to post-mentorship (mean 7.2, SD=1.9 to 9.3, SD=0.8; mean difference=2.17, 95% CI 1.85–2.51). Moderate positive correlation between knowledge and self-efficacy at post-mentorship (r=0.47, p<0.001). | First study to assess the impact of a practice-based mentorship programme on neonatal resuscitation knowledge and self-efficacy among nurses and midwives in Rwanda. Length of practice experience and education level were not significantly associated with neonatal resuscitation outcomes, suggesting mentorship was the key factor. No control group; no long-term retention assessment post-mentorship. Only three of 30 districts sampled, limiting generalisability. |
| Odongkara et al. (2020) | Uganda  (Low-income) | To assess the effect of adding video-debriefing to standard Helping-Babies-Breathe (HBB) simulation-based training on neonatal resuscitation knowledge and skills attainment and retention among skilled birth attendants | Cluster-randomised controlled trial | 86 Midwives, Nurses, Doctors, Clinical Officers.  (Health facilities) | Helping Babies Breathe (HBB) 2nd edition with video-debriefing (intervention) vs standard HBB 2nd edition (control) | Norwegian Government through NORHED (Survival Pluss project, Makerere University); University of Bergen, Norway; Sachs’ Children and Youth Hospital, Stockholm, Sweden | Intervention arm: standard HBB 2nd edition training (2 days: integrated lectures, demonstrations, group practice in threes with NeoNatalie manikin) plus video-debriefing of simulation sessions using the SHARP debriefing tool (Set learning objectives, How it went, Address concerns, Review learning points, Plan ahead) and the Objective Assessment of Debriefing (OSAD) tool. Control arm: standard HBB 2nd edition training only (same 2-day curriculum without video-debriefing). Knowledge assessed by standardised HBB MCQs; skills assessed by BMV, OSCE-A and OSCE-B checklists at pre-test, post-test, 1, 3, and 6 months. | June 2018 – January 2019 (training in June 2018; 6-month follow-up) | 6 months | (1) Knowledge and skills attainment (immediate post-training scores), (2) Knowledge and skills retention (over 6-month follow-up at 1, 3, and 6 months post-training) | Adding video-debriefing to standard HBB training significantly improved combined knowledge and skills attainment post-training (adjusted mean difference: 5.34; 95% CI 0.40–10.28, p=0.034) and retention over 6 months (adjusted mean difference: 2.97; 95% CI 1.52–4.41, p<0.001) after adjusting for confounding and clustering. Factors that reduced competence retention: monthly resuscitation of ≥1 neonate and >5 years in service. | Video-debriefing enhanced competence attainment and retention over 6 months. Approximately 80% of participants had no prior HBB training. Frequent assessments at close intervals may have influenced retention through revision pressure. Senior skilled birth attendants (>5 years) demonstrated inferior retention, possibly due to slower uptake of new practices. 95.3% follow-up rate at 6 months. Authors recommend further mixed-methods research with cost-benefit analysis to justify scale-up. |
| Patterson et al. (2023) | Democratic Republic of the Congo (Low-income) | To address the gap in understanding of resuscitation practices following HBB 2nd edition training through a detailed analysis of provider care during observed resuscitations | Secondary analysis of an interventional trial (pre-post observational study) | 59 providers (45 midwives, 14 physicians) at 3 health facilities in Kinshasa (annual births ranging from 1,051 to 4,248)(Hospitals) | Helping Babies Breathe (HBB) 2nd edition | University of North Carolina at Chapel Hill, USA; University of Kinshasa, DRC; Laerdal Medical, Norway; RTI International, USA; American Academy of Pediatrics, USA | One-day in-service HBB 2nd edition workshops conducted by experienced in-country HBB master trainers in French. Skills practice in pairs using NeoNatalie manikin, upright bag and mask, and penguin suction device. HBB action plans posted in delivery wards. Providers practiced skills with OSCEs approximately once monthly. Battery-operated NeoBeat HR meter used on non-breathing newborns. Observational data collected via Liveborn tablet app by trained research staff. | October 2018 – July 2019 (approximately 9 months) | During implementation period only (convenience sample of observed resuscitations) | (1) Knowledge (post-knowledge check, 19 items), (2) Skills (BMV skills checklist, 14 items; OSCE A, 14 items), (3) Practice (order of resuscitation steps, timing and duration of drying/stimulation, suctioning, and BMV via direct observation) | (1) Post-training median knowledge check score 79% (IQR 74–87); BMV skills check improved from median 36% (IQR 21–57) pre-training to 79% (IQR 68–93) post-training; OSCE A improved from 43% (IQR 29–57) to 79% (IQR 71–86). (2) Data collected from 2,592 observed liveborn neonates: providers dried/stimulated before suctioning in 97% and suctioned before ventilating in 100% of cases. Only 19.7% of newborns not breathing well by 60 seconds ever received BMV. Median time to BMV initiation was 347 seconds (>5 minutes); no cases initiated within the Golden Minute. During 81 resuscitations involving BMV, median 132 seconds spent drying/stimulating and 98 seconds suctioning. 72% of initial BMV episodes lasted <60 seconds. | HBB-trained providers followed correct order of resuscitation steps but frequently failed to initiate BMV. When BMV was initiated, it was substantially delayed and interrupted by repetitive stimulation and suctioning. Providers overused suctioning (56.7% of breathing-well newborns suctioned). Pragmatic implementation with monthly OSCE practice only. Research staff underwent rigorous training for observational data collection using Liveborn app. Unable to compare practices before and after HBB training (no pre-training practice data). |
| Patterson et al. (2025) | Democratic Republic of the Congo (Low-income) | To evaluate the impact of heart rate-guided basic resuscitation compared to Helping Babies Breathe on neonatal outcomes and resuscitation practices | Pre-post interventional trial | 59 providers trained in HBB (control period) and 56 providers trained in HR-guided basic resuscitation (intervention period). Midwives were the primary providers of newborn resuscitation care.(3 urban health facilities in Kinshasa, DRC) | HBB 2nd edition (control) vs HR-guided basic resuscitation (intervention): adapted HBB action plan and flipchart to include HR assessment to prompt initiation and improvement of ventilation | University of North Carolina at Chapel Hill, USA; University of Kinshasa, DRC; Laerdal Medical, Norway; RTI International, USA; American Academy of Pediatrics, USA | Control period: standardised HBB 2nd edition training with skills practice and assessments in French; providers trained to place NeoBeat HR meter on non-breathing newborns; monthly manikin practice. Intervention period: additional one-day training in HR-guided basic resuscitation using adapted HBB action plan with HR assessment at <100 bpm to prompt ventilation initiation; pilot-tested in Nepal; monthly manikin practice with HR-guided OSCEs. Observational data collected via Liveborn app synchronised with NeoBeat HR data. | Control (HBB): October 2018 – July 2019 (9 months). Intervention (HR-guided): August 2019 – June 2020 (10 months) | During both implementation periods (convenience sample of observed resuscitations) | (1) Knowledge and skills (HR-guided basic resuscitation assessments: knowledge check, OSCE A, OSCE B), (2) Effective breathing at 3 minutes after birth (primary outcome), (3) Time to effective breathing, (4) HR ≥100 bpm at 3 minutes, (5) Time to HR ≥100 bpm, (6) Time to BMV initiation, (7) APGAR scores, (8) Death before discharge, (9) Perinatal mortality (total stillbirths + death before discharge) | HR-guided training: median post-knowledge check 95 (IQR 90–100); post-OSCE A 92.3% (IQR 84.6–100); post-OSCE B 81.8% (IQR 77.3–86.4). Data collected from 10,906 livebirths (6,185 HBB; 4,721 HR-guided) with 1,284 observed with NeoBeat data. (1) No difference in effective breathing at 3 minutes (aRR 1.08 [95% CI 0.80–1.45]). (2) Time to HR ≥100 bpm decreased by 7.69 seconds (p=0.02). (3) Among 145 receiving BMV, time to BMV decreased by 64.5 seconds (p<0.001; median 327 to 283 seconds). (4) Death before discharge increased (aRR 1.59 [95% CI 1.13–2.23]). (5) Stillbirth misclassification rate decreased from 33.3% during HBB to 5.9% in HR-guided resuscitation. | During HR-guided resuscitation, providers were more efficient in responding to non-breathing neonates; however, BMV initiation remained well beyond the Golden Minute (median 283 seconds). Increased death before discharge may reflect resuscitation of newborns previously presumed stillborn who had higher baseline mortality risk. Pre-post design limits causal inference; on-going training and simulation practice may have influenced changes. Respiratory status subjectively determined by research staff. More than one-third of 5-minute Apgar scores missing during control phase. No data collected beyond birth hospitalisation. |
| Clark et al. (2023a) | Nepal  (Lower-middle-income) | To report on the implementation and changes in newborn outcomes during the Safa Sunaulo Nepal (SSN) program for scaling up newborn resuscitation capacity-building and skill retention in Gandaki Province, Nepal | Prospective cohort study | 43 facility-based trainers cascading resuscitation skills to 425 medical personnel (physicians, nurses, midwives) at 12 health facilities (7 level I hospitals, 5 level II hospitals) in Gandaki Province(Hospitals) | HBB training through the Safa Sunaulo Nepal (SSN) program of scaling up neonatal resuscitation training and skill retention | Latter-day Saint Charities (LDSC) | Two-day HBB Training-of-Trainer (ToT) course facilitated by 9 Nepali trainers and 4 US-based trainers. Facility-based trainers cascaded HBB training to staff. Skill retention strategies included: daily low-dose, high-frequency bag-and-mask ventilation practice, post-resuscitation self-evaluation forms, weekly group review sessions, monthly resuscitation debriefings led by external mentor, refresher training. Single external mentor provided on-site coaching, reviewed practice logbooks and delivery logs, and liaison with hospital administration. | March 2018 – November 2019 (21 Nepali months) | During implementation period only | (1) Intrapartum/fresh stillbirths, (2) Neonatal deaths within 24 hours of birth, (3) Neonatal deaths after 24 hours of birth, (4) Use of bag-and-mask ventilation on non-breathing newborns, (5) Number of sick newborns discharged from the maternity unit (proxy for all-cause newborn morbidity) | Data collected from 33,417 deliveries (23,820 vaginal, 9,597 caesarean) across 12 health facilities. (1) No significant change in intrapartum stillbirths (IRR=1.000, p=0.569). (2) Significant declining trend in neonatal deaths within 24 hours (IRR=0.993, p=0.044), a 0.7% decrease per unit change in time. (3) No significant change in neonatal deaths after 24 hours (IRR=1.130, p=0.412). (4) No significant change in bag-and-mask ventilation use (IRR=1.000, p=0.104). (5) Significant declining trend in sick newborns discharged (IRR=0.996, p<0.001), a 0.4% reduction per unit change in time. | Findings consistent with a previous implementation in Western Nepal. Monthly monitoring with data quality checks by a dedicated external mentor. Low-cost cascade training model with a single external mentor supervising multiple facilities. Self-evaluation survey utilisation was poor; practice corners with logs only scaled to two-thirds of hospitals. Facilitated practice sessions with mentor/trainer were more consistent than spontaneous provider practice. No control/comparison group; unable to confirm whether outcomes were solely attributable to the programme. Seasonal variation in adverse birth outcomes may have biased estimates. |
| Clark et al. (2023b) | Nepal  (Lower-middle-income) | To report on change in newborn outcomes after an evidence-based intervention for scaling up neonatal resuscitation training and skill retention implementation | Prospective cohort study | 443 facility-based trainers from 191 health facilities trained via ToT; 6,389 providers trained across 87 actively mentored facilities in 5 provinces(Health facilities ranging from local-level to tertiary-level, public and private) | HBB training through the Safa Sunaulo Nepal (SSN) program of scaling up neonatal resuscitation training and skill retention | Latter-day Saint Charities (LDSC) | HBB Training-of-Trainer (ToT) courses held in Nepali and English. Facility-based trainers cascaded HBB training to staff including new staff and refresher sessions. Skill retention strategies: functional practice corners with logs, low-dose high-frequency practice, self-evaluation forms, team review meetings, refresher training, on-site coaching by external mentor, and liaison with hospital administration. Each geographic area had a full-time external mentor supporting 12–18 facilities. Programme transitioned to internal mentoring with appointment of focal persons at facilities. | December 2017 – November 2021 (48 months across 5 provinces with variable implementation durations) | During implementation period only | (1) Intrapartum stillbirths, (2) Neonatal deaths within 24 hours of birth, (3) Sick newborns transferred from the maternity unit (proxy for all-cause morbidity), (4) Use of bag-and-mask ventilation | Data collected from 315,673 deliveries across 87 facilities in 5 provinces. (1) Significant reduction in intrapartum stillbirths in 4 of 5 provinces: Lumbini (−72.85%, p=0.0013), Gandaki (−57.14%, p=0.0366), Madhesh (−56.15%, p<0.001), Karnali (−86.84%, p=0.0001); not significant in Bagmati (p=0.423). (2) Significant reduction in neonatal deaths within 24 hours in 3 provinces: Lumbini (−60.31%, p=0.0144), Madhesh (−56.42%, p<0.001), Karnali (−89.47%, p=0.00006). (3) Significant reduction in sick newborn transfers in 3 provinces: Lumbini (−76.92%, p=0.0145), Gandaki (−59.57%, p=0.0313), Madhesh (−30.74%, p<0.001). | Programme faced significant barriers due to nationwide COVID-19 mobility restrictions (March–July 2020, April 2021 onward) causing sporadic cascade training and lower provider participation in affected provinces. Bagmati province (hosting capital Kathmandu) had higher baseline capacity and showed non-significant improvements. Karnali province (most remote, poorest) showed highest reductions, likely due to bridging existing skill and equipment gaps. Cascade training approach was low-cost, avoiding travel costs and daily subsistence allowances. No control/comparison group; unable to establish causality. Misclassification and underreporting of fresh stillbirths were widespread, requiring delivery log review. |
| Spies et al. (2022) | India (Lower-middle-income) | To assess the improvement and retention of resuscitation skills of nurses, midwives, and birth attendants in 2 birth centres serving 60 villages in rural India using high-frequency, low-dose training | Cluster randomised controlled trial | 48 nonphysician care providers (28 intervention, 20 control): village healthcare workers, Accredited Social Health Activists (ASHA), auxiliary nurse midwives at 2 birth centres in Mahbubnagar District, Telangana(Birth centres serving 60 villages) | Navjat Shishu Suraksha Karyakram (NSSK): adapted Helping Babies Breathe (HBB) and Essential Newborn Care (ENC) programme from the Indian Ministry of Health and Family Welfare | Baylor University, Texas, USA; University of Connecticut, USA; Neonatal Intensive Care and Emergency (NICE) Foundation, Telangana, India; Laerdal Foundation (grant #40185) | Control group: one-time workshop of NSSK (HBB and Essential Newborn Care) with lecture, video, and hands-on education; no repeat sessions. Intervention group (NSSK-Plus): same initial workshop plus train-the-trainer model with 3 local expert trainers per site providing monthly refresher courses using specifically developed video teaching modules in Telugu; quarterly researcher-led workshops with augmented education (equipment maintenance, Apgar scoring, delayed cord clamping, lactation, skin-to-skin care). All providers received resuscitation kits (bags, masks, suction devices, blankets, mannequin, HBB flip chart in Telugu, tablets with HBB videos). | January 2017 – October 2017 (8 months; originally planned for 12 months but shortened due to closure of one birth centre) | 1 month and 8 months post-initial training | (1) Knowledge retention (15-item MCQ derived from HBB Knowledge check at baseline, 1 month, and 8 months), (2) Skills (standardised OSCE at baseline and 6 months), (3) Birth outcomes (resuscitation initiation, successful resuscitation, stillbirths, neonatal deaths, skin-to-skin placement within 2 hours, lactation within 24 hours, temperature recording, transfer to higher-level care) | (1) Significant difference in 8-month posttest knowledge scores between groups (intervention mean rank 19.4 vs control mean rank 10.3, p=0.003). Control group showed significant immediate post-test improvement (Z=−3.47, p=0.001) but intervention group did not (Z=−0.91, p=0.36). (2) Data from 511 births (205 control, 306 intervention): significantly lower need for resuscitation in intervention group (5.9% vs 18.0%, p<0.001). Resuscitation success rate not significantly different (94.6% control vs 88.9% intervention, p=0.590). No significant differences in stillbirths (p=0.653), neonatal deaths (p=0.680), skin-to-skin within 2 hours (p=0.745), or lactation within 24 hours (p=0.524). | First proof of concept for high-frequency, low-dose education model in rural India. Study was underpowered due to unexpected political redistricting of Mahbubnagar District (divided into 4 districts) limiting access to 2 of 4 original birth centres. Post hoc power analysis showed only 7.4% power for detecting difference in knowledge score changes. Anecdotally, intervention group healthcare workers had greater skill retention and voiced higher confidence. Train-the-trainer model demonstrated as cost-efficient retraining methodology. Nurse midwives had significant influence in villages due to proximity and visibility. |
| Stordal et al. (2020) | Tanzania (Lower-middle-income) | To study whether the reduction in perinatal mortality after introduction of Helping Babies Breathe was sustained, and whether resuscitation skills changed with continued frequent training over a five-year period | Prospective cohort study | Midwives (primary providers of newborn resuscitation) at Haydom Lutheran Hospital, a rural hospital in Manyara region with 250–400 deliveries per month and catchment area of over 2 million inhabitants(Hospital) | HBB training through the Safer Births program | Laerdal Medical AS, Norway | HBB training initially implemented 2009–2012. The Safer Births programme (starting 2013) continued low-dose, high-frequency skills training: simulation on manikins assisted by local trainers with HBB facilitator training; visiting instructors from Tanzania and Norway participated regularly; trainings intended weekly with some variation. Heart rate displayed via dry-electrode ECG sensor (Newborn Resuscitation Monitor, Laerdal Global Health) during resuscitation; ventilation parameters collected via bag sensors but not displayed to providers. | July 2013 – June 2018 (5 years) | During implementation period only | (1) Early neonatal mortality (death before 24 hours), (2) Perinatal mortality (fresh stillbirths + early neonatal mortality), (3) Clinical performance: time from birth to start of BMV, duration of first BMV sequence, fraction of time used for BMV, total duration of BMV, ventilation frequency, tidal volumes, mask leak | Data collected from 19,571 births (18,997 live births). Deliveries decreased by 30% over the period (likely due to introduction of user fees). (1) Early neonatal mortality averaged 8.8/1,000 live births with no significant change unadjusted (RR per year 1.11, 95% CI 1.00–1.24); adjusted for vulnerability factors no significant reduction (RR per year 0.95, 95% CI 0.84–1.07). (2) Perinatal mortality: significant annual decrease of 16% after adjustment for vulnerability factors (RR per year 0.84, 95% CI 0.76–0.94, p=0.002). (3) Duration of first BMV sequence increased from median 11 to 32 seconds (p<0.001); BMV during first 60 seconds increased from median 24 to 48 seconds (p<0.001); ventilation frequency decreased from >60 to 44–48 breaths/min (p<0.001); tidal volumes increased (p<0.001); mask leak decreased (p=0.001). | First study to report long-term changes in actual ventilation performance after HBB implementation. Introduction of user fees (12–30 USD) caused a 30% decrease in hospital deliveries, selecting for more vulnerable newborns (proportion with initial HR <100 bpm increased from 8% to 22%). Adjusted analyses accounted for this selection bias. One-third of early neonatal deaths occurred in newborns who did not receive BMV, suggesting causes beyond intrapartum hypoxia (e.g., prematurity, sepsis). Residual confounding cannot be fully ruled out in this observational study. Further reduction in neonatal mortality requires comprehensive approach to increase facility births and address multiple causes of death. |
| Tadesse et al. (2021) | Ethiopia (Low-income) | To examine the impact of low-dose, high-frequency (LDHF) training for introducing a nurse-led neonatal advanced life support (NALS) service on delivery room neonatal outcomes in a tertiary care hospital in Ethiopia | Retrospective cohort study | 11 providers (10 NICU nurses and 1 midwife with BSc degrees and ≥2 years bedside experience), selected from existing hospital staff at St. Paul’s Hospital Millennium Medical College (SPHMMC), Addis Ababa(Tertiary referral hospital with >12,000 annual deliveries) | Neonatal Advanced Life Support (NALS) programme building on AAP Helping Babies Survive programmes, Neonatal Resuscitation Program, S.T.A.B.L.E. programme, and AWHONN competency-based orientation | Wax and Gold Inc. (US-based 501(c)3 non-profit organisation) | 12-month longitudinal LDHF curriculum: 110 hours of didactic presentations and simulation-based training, 120 hours of hands-on practicum in the labour and delivery unit with case review, 20 hours of supplemental lectures on quality improvement and data management, followed by 6 months of intermittent bedside clinical mentorship by US and Ethiopian neonatal care experts. Training conducted in the same clinical environment where NALS team would practice. Standardised data collection form developed and validated; computerised MySQL database maintained. T-piece resuscitators and plastic wrap for thermoregulation introduced. | December 2016 – June 2017 (service rollout); data collection June 2016 – March 2019 (approximately 33 months including pre-implementation) | Pre-implementation (June–September 2016) compared with 5 post-implementation periods (June 2017–March 2019; 3–22 months after implementation) | (1) Neonatal deaths in the inpatient labour and delivery unit (LDU), (2) Rate of NICU admission from the inpatient LDU, (3) Proportion of neonates with normal axillary temperature (36.5–37.5°C) within the first hour of life, (4) Qualitative analysis: semi-structured interviews with 20 key stakeholders | Data collected from 14,067 deliveries (2,066 pre-implementation; 12,001 post-implementation). (1) Significant sustained decrease in LDU neonatal deaths from 3.5% (pre) to 1.0% (initial post) to 0.3–0.5% (sustained, p<0.001). (2) NICU admission rate: initial non-significant change (22.8% to 21.2%, p=0.6), followed by significant sustained decrease to 7.8–9.0% (p<0.001). (3) Normal axillary temperature improved from 46.2% to 87.8% (p<0.01), then to 99.8%. (4) Qualitative analysis: NALS team reported increased confidence and pride; NICU providers and administrators praised outcomes, improved workflow, and desired expansion of training. | First hospital in Ethiopia with certified NALS providers responsible for all high-risk deliveries. Two cohorts of NALS providers certified to date. Training pace set to slowest learner; conducted in actual clinical practice environment. Programme sustainability supported by train-the-trainer model with first cohort members training second cohort. Limitations: causes of death not recorded; neonatal temperatures not recorded pre-implementation; no concurrent QI projects in LDU during implementation. Staff resistance to change addressed through group meetings presenting outcome data. Collaboration initiated with Ministry of Health to pilot NALS service in health centres and non-referral hospitals. |
| Taneja et al. (2021) | India (Lower-middle-income) | To design and implement the Care Around Birth approach to synergise evidence-based technical interventions with quality improvement processes, respectful maternity care, and health system strengthening to improve intrapartum and immediate postpartum quality of care | Pre-post intervention study with external evaluation | 1,410 staff nurses and auxiliary nurse-midwives (ANMs) at 141 high-caseload public health facilities across 26 high-priority districts in 6 states (Delhi, Haryana, Himachal Pradesh, Jharkhand, Punjab, Uttarakhand)(District hospitals, community health centres, referral hospitals, subdivisional hospitals, primary health centres) | Care Around Birth approach under USAID Vriddhi (Scaling up RMNCH+A Interventions) Project: 14 technical interventions adapted from national guidelines | USAID; IPE Global; Ministry of Health and Family Welfare (MOHFW), Government of India; 6 state governments | Experiential trainings using adult learning principles: simulations, demonstrations, case studies, role playing, participatory methods. Centralised 2-day district-level training followed by on-site low-density, high-frequency training sessions for facility staff saturation. On-site monthly mentoring visits with drills, checklists, job aids, peer-to-peer learning, and data-for-action reviews. Facility-level quality improvement (QI) teams using plan-do-study-act approach. District- and state-level experience-sharing platforms. Respectful maternity care (RMC) integrated into training, mentoring, and QI. Data management built on USAID Collaborating Learning and Adapting framework. | January 2016 – December 2017 (24 months) | Baseline: October–December 2015; endline external evaluation: October–December 2017 (conducted across sample of 51 facilities with direct observation of 399 deliveries and 392 beneficiary interviews) | (1) Labour room environment (amenities, equipment, protocols, infection control), (2) Provider competency (knowledge and skills via vignette, knowledge tests, and simulation-based OSCE for partograph use, active management of third stage of labour, essential newborn care, newborn resuscitation, postnatal monitoring, infection prevention, newborn vaccination), (3) Labour room practices (partograph use, oxytocin administration, essential newborn care, resuscitation, postnatal monitoring), (4) Provision and experience of care (endline only) | Data from 463,713 mothers and 458,152 newborns across 141 facilities. (1) Delivery trays available from 66% to 98%; newborn trays from 55% to 98%. (2) Competency scores >80%: essential newborn care improved from 7% to 70%; newborn resuscitation from 5% to 82%. (3) Partograph use improved from 29% to 61% (p<0.001 at key intervention phases); oxytocin within 1 minute from 35% to 93% (p<0.001); delayed cord clamping from 35% to 90%; newborns successfully resuscitated from 71% to 96%; postnatal monitoring of mothers within 6 hours from 33% to 86% (p<0.001). Maximum improvement observed after mentorship and establishment of QI teams. (4) At endline: 100% women received oxytocin; breastfeeding initiated within 1 hour in 96%; 97% satisfaction among clients. | Staggered implementation built health system capacity to absorb change and ensured facility readiness for QI approaches. Maximum impact observed after combination of mentoring and QI team establishment, not training alone. QI teams used predefined data elements across multiple indicators simultaneously rather than addressing one improvement aim at a time. Approach integrated maternal and newborn care by designing training to follow sequence of birth events. State-level scale-up achieved in Punjab and Delhi; approach integrated into national LaQshya labour room QI initiative. Limitations: different methodology for baseline and endline assessments; no control districts; provision and experience of care measured at endline only (possible Hawthorne effect); short implementation period for some interventions limits sustainability assessment. |
| Teh et al. (2024) | Malaysia  (Upper-middle-income) | To evaluate the retention of knowledge, skills, and competency following Neonatal Resuscitation Programme (NRP) training among house officers | Before-after study | 34 house officers (doctors)  (Hospital) | Neonatal Resuscitation Programme (NRP) | Not reported | Reading materials (NRP textbook), practical resuscitation skills training using low-fidelity simulation manikins (initial steps, bag-mask ventilation, intubation, cardiac massage, umbilical vein cannulation), leadership and teamwork training, Objective Structured Clinical Examination (OSCE) A and B assessment | Not reported (NRP made a key performance indicator [KPI] in 2016) | 3, 6, and 9 months | (1) Knowledge (multiple-choice questionnaire [MCQ]), (2) Skills (bag-mask ventilation, intubation, initial steps using NRP checklists), (3) Competency (OSCE A and OSCE B performance) | (1) Significant reduction in theoretical knowledge over time (p<0.05). (2) No significant decline in initial step skills (p>0.05); significant decline in bag-mask ventilation (p<0.05) and intubation skills (p<0.05). (3) Significant decrease in OSCE A (p<0.05) and OSCE B (p<0.05) performance. | Time lapse after training and rotation to non-paediatric departments reduced staff exposure to neonatal resuscitation and hindered sustainability. No comparator group (pre-intervention comparison only). |
| Tosif et al. (2020) | Solomon Islands  (Lower-middle-income) | To determine the impact on skills and knowledge amongst healthcare workers following Early Essential Newborn Care (EENC) training, and characterise provider attributes affecting retention | Pre-post study | 53 healthcare workers (23 nurses, 15 doctors, 8 midwives, 7 nurse aides) across 5 hospitals  (Hospitals) | EENC (Early Essential Newborn Care) Program | Not reported | Two-day on-site coaching programme with low participant-to-facilitator ratio (6:1), participatory style without didactic teaching, repeated practice using EENC skills checklists for breathing and non-breathing baby scenarios, establishment of quality improvement team within 3 months, refresher training at evaluation. | March 2015-Dec 2017 | 12 months | (1) Knowledge, (2) Skills | (1) Significant increase in written knowledge scores from 44% to 89% post-coaching (p<0.001); declined to 61% at evaluation (p<0.001), still 17% above baseline. (2) Skills in breathing baby scenario increased from 20% to 95% post-coaching (p<0.001); declined to 82% at evaluation (p<0.001). Non-breathing baby scenario: 63% to 86% post-coaching; declined to 72% at evaluation (p<0.001). Nurse aides and midwives had least reduction in scores at evaluation. | EENC coaching improved knowledge and skills but declined over time without complementary strategies. Healthcare workers who used skills in regular practice had higher scores. No comparator group (pre-intervention comparison only). |
| Umoren et al. (2021) | Nigeria and Kenya  (Lower-middle-income) | To assess the impact of mobile virtual reality (VR) simulations (electronic Helping Babies Breathe [eHBB]) or video for maintenance of neonatal resuscitation skills in healthcare workers in resource-scarce settings | Randomised controlled trial | 274 nurses and nurse-midwives (VR group n=91; video group n=95; control group n=88)  (Hospitals) | HBB training | The US | One-day in-person HBB second edition course, followed by 6-month digital refresher: VR group received eHBB VR simulation (three interactive 3D scenarios) plus digital HBB provider guide; Video group received neonatal resuscitation video plus digital guide; Control group received digital guide only. All groups had access to low-dose high-frequency (LDHF) manikin practice at facility HBB corners. | Dec 2018-Feb 2020 | 6 months | (1) Knowledge, (2) Skills | No significant differences in 6-month pass rates across groups for BMV (VR 28%, video 25%, control 22%, p=0.71), OSCE A (VR 76%, video 76%, control 72%, p=0.78), or OSCE B (VR 62%, video 60%, control 49%, p=0.18). Greater retention of BMV skills at 6 months in VR group (−15%, p=0.10) vs video (−21%, p<0.01) and control (−27%, p=0.001). OSCE B pass rates higher at 3 and 6 months in VR group compared with immediate post-course. 95% of VR and 98% of video respondents would use their intervention again. | eHBB VR training was highly feasible and acceptable to healthcare workers in low-income to middle-income countries. VR may provide additional support for neonatal resuscitation skills retention compared with other digital interventions. Three-arm randomised controlled trial with 6-month follow-up. |
| Umunyana et al. (2020) | Rwanda  (Low-income) | To examine the effect of complementing provider training with clinical mentorship and quality improvement as outlined in the second edition of the HBB material | Before-after mixed methods study | 772 Healthcare providers across 172 facilities  (Hospitals and health centres) | HBB 2nd edition training with clinical mentorship and quality improvement | USAID | HBB training, low dose high frequency (LDHF) method, clinical simulations, quality improvement activities, mentorship activities (mentoring skills and refresher training in HBB) | January 2016 – June 2018 (28 months, phased across 10 districts) | Up to 1 year post-training (mentorship visits); 5 months follow-up of competent providers | (1) Knowledge and skills (HBB OSCE B pre/post training and during mentorship visits), (2) Neonatal outcomes (successful resuscitation of newborns not breathing at birth, asphyxia admissions to neonatal intensive care, deaths due to asphyxia, deaths within 30 min of birth, fresh stillbirths) | (1) Improved health worker knowledge and skills 60% competence on the first mentorship visit, and 100% by the sixth, (2) Neonatal admissions due to asphyxia per 1000 live births reduction of 12.9% (95%CI = 8.3–17.5%), Neonatal deaths due to asphyxia reduction of 4.2% (95%CI =0.2–6.3%), Fresh stillbirths per 1000 deliveries reduction of 6.2% (95%CI = 2.1–11.4%), no significant in deaths within 30 min of birth (p = 0.06). | Large-scale implementation across 172 facilities (33% of Rwanda population). Mentorship, quality improvement, and continuous learning were critical components. Clinical mentorship emerged as the most important element. No comparator group (pre-intervention comparison only). |
| Vadla et al. (2022b) | Tanzania  (Lower-middle-income) | To describe changes in clinical resuscitation performance and perinatal outcomes after introducing a novel simulator (NeoNatalie Live) and local champions to facilitate ongoing Helping Babies Breathe (HBB) simulation training | Prospective before-after (2 phases) clinical observational study | 18–22 midwives (number varied due to high staff turnover)  (Hospital) | HBB training | Norway | Phase 1 (13 months): HBB second edition with NeoNatalie Live simulator for mainly self-guided individual skill training. Phase 2 (11 months): local champions (4 dedicated junior midwives) appointed to facilitate weekly training including individual skill and scenario team training. | September 2015 – August 2018 (36 months: 12-month baseline, 13-month phase 1, 11-month phase 2) | During implementation period only | (1) Practice/performance (proportion of newborns receiving stimulation, suction, and/or bag-mask ventilation [BMV] immediately after birth; time from birth to first ventilation), (2) Perinatal mortality (fresh stillbirth [FSB], 24-hour neonatal mortality, admissions to neonatal care unit) | Data from 10,481 births. (1) Significant decrease in time from birth to first ventilation from median 118 to 101 seconds (p=0.007); proportion of midwives trained in last 7 days increased from 34% to 71%. (2) No significant change in risk for perinatal deaths after adjustment for risk factors (p=0.898), despite increased population vulnerability. | Training frequency increased substantially only after introduction of local champions. High staff turnover remained a challenge. Pauses during ventilation decreased significantly. No comparator group (pre-intervention comparison only). |
| Vadla et al. (2024) | Tanzania  (Lower-middle-income) | To decrease time from birth to ventilation and document potential changes in perinatal outcomes after implementation of a quality improvement (QI)/simulation intervention (the Golden Minute Campaign) | Prospective 4-year observational quality improvement study | Healthcare providers in the labour ward  (Hospital) | The Golden Minute Campaign, HBB training | Laerdal Medical AS, Norway | QI/simulation intervention including: Train-the-trainer simulation course (EuSim Level 1) for local champions, QI monthly meetings with clinical data review, monthly scenario team simulation training targeting identified gaps, LDHF individual skill training, clinical debriefing after resuscitation, weekly morning reports on clinical data, annual 1-day HBB course. | 4 years | 1 year and 2 years after implementation | (1) Practice (Time from birth to first ventilation attempt,  Proportion of non-breathing newborns ventilated within the Golden Minute (60 seconds),  Proportion of newborns receiving stimulation, suction, and/or bag-mask ventilation, and Healthcare workers' frequency of training) (2) Neonatal outcomes (FSB, 24-hour newborn deaths, and admissions to the neonatal care unit ) | Data collected from 12,678 deliveries. (1) Practice (Significant increase in timely ventilation (p= 0.001), Significant reduction in time to ventilation (p=0.001), Significant Increased ventilation of nonbreathing newborns (p=0.001), (2) Significant reduced in FSB (p=0.013) during the intervention period, No statistically significant changes in perinatal mortality for all births in unadjusted (P = 0.468) or adjusted analyses (P = 0.182) | Combination of QI efforts and trained local champions, strongly supported by hospital management, was crucial. Improvements partially reversed during 2-year post-intervention period, highlighting need for continuous simulation-based training. No comparator group (pre-intervention comparison only). |
| Vadla et al. (2022a) | Tanzania  (Lower-middle-income) | To describe changes in staff participation, skill-training frequency, and simulated ventilation quality before and after the introduction of local motivators to facilitate HBB training | Prospective observational study | 30 Midwives  (Hospital) | HBB training, NeoNatalie Live simulator, and local motivators | Norway | Phase 1 (12 months): self-guided individual skill training using NeoNatalie Live simulator with automated feedback. Phase 2 (11 months): local motivators (4 dedicated junior midwives) appointed to encourage training, provide technical support, and arrange scenario team training. Weekly bag-mask ventilation practice encouraged. | Sept 2016-Aug 2018 | Continuous automated monitoring over 2-year study period | (1) Staff participation (percentage of midwives training), (2) Training frequency (number of skill-training sessions), (3) Simulated ventilation quality (percentage of well-done feedback, valid ventilations), (4) Monthly trainings per midwife | (1) Staff participation increased from median 43% to 74% (p=0.016). (2) Training frequency increased from 688 to 8,451 sessions after introduction of local motivators (p<0.001). (3) Training quality improved from 75% to 91% well-done feedback (p<0.001); valid ventilations from 98% to 100% (p<0.001). (4) Monthly trainings per midwife increased from median 2.3 to 26.4 (p<0.001). | Local motivators were the key driver of increased training frequency and participation. NeoNatalie Live simulator with automated feedback may have contributed to training quality. Conducted in a low-resource setting with high staff turnover. No comparator group (pre-intervention comparison only). |
| Wilson et al. (2020) | Tanzania  (Lower-middle-income) | To assess the efficacy and viability of implementing Helping Babies Breathe (HBB) on a small budget in two of the largest delivery centres in Zanzibar, Tanzania | Mixed methods quality improvement study with repeated measures | 59 midwives and nurses (55 nurse-midwives, 4 registered nurse officers)  (Hospitals) | HBB training | The US | Train-the-trainer model with 3-day HBB second edition training (hands-on demonstration, return demonstration, simulation using NeoNatalie with case scenarios, small-group teamwork). Followed by 6 months of supportive supervision of real-time deliveries (130 observations), practice stations, birth record data collection, and quality improvement outline. | Not reported | 6 months | (1) Knowledge, (2) Skills, (3) Bag-Mask Skill | Significant improvement in knowledge (p<0.001), bag-mask skills (p<0.001), and OSCE scores (p<0.001) immediately post-training. Scores maintained or improved at 6 months: knowledge 15.64 to 16.29, OSCE 18.02 to 19.13, bag-mask skills 12.84 to 12.87. During 130 supportive supervision observations, 18 neonates (14%) required intervention; all survived the Golden Minute. Total budget: 9,015.50 USD. | Low-budget implementation demonstrates feasibility. Train-the-trainer strategy with local master trainers was effective. Supportive supervision observations successfully translated HBB knowledge and skills into clinical practice. Investment from local government and local leadership was critical for sustainability. No comparator group (pre-intervention comparison only). |
| Xu et al. (2022) | China  (Upper-middle-income) | To investigate the impact of weekly multidisciplinary in-situ simulation training (MIST) on neonatal resuscitation in reducing the incidence of neonatal asphyxia and related morbidities | Retrospective before-after intervention study | 225 active participants (1,503 participant-counts): physicians, midwives, nurses, medical trainees, and obstetricians  (Hospitals) | Multidisciplinary In-situ Simulation training (MIST) based on NRP program | Not reported | Weekly in-situ simulation in delivery room facilitated by 2 instructors and performed by 3 healthcare providers from obstetric and NICU units. Each session included briefing with learning objectives, simulated resuscitation on manikin (81 scenarios over 2 years), and structured debriefing with designated observers. Scenarios based on NRP algorithm. | Jan 2017-Dec 2020 | Immediately post-training only | (1) Neonatal asphyxia incidence, (2) Severe asphyxia incidence, (3) Hypoxic-ischaemic encephalopathy (HIE) incidence, (4) Meconium aspiration syndrome (MAS) incidence | Data from 29,759 live births. Significant decreases in neonatal asphyxia (0.84% to 0.64%, p=0.045), severe asphyxia (0.14% to 0.06%, p=0.029), HIE (0.10% to 0.01%, p=0.003), and MAS (0.19% to 0.09%, p=0.014) after commencement of weekly MIST. | Adopting cultural concept that errors can occur during resuscitation and creating a safe learning environment enhanced participation. Reused clinical equipment for training, making it economically sustainable. Multidisciplinary collaboration between NICU and obstetric units was essential. No comparator group (historical pre-intervention comparison). |

*Abbreviations: AAP, American Academy of Pediatrics; AHA, American Heart Association; BMV, bag-mask ventilation; ECEB, Essential Care for Every Baby; FSB, fresh stillbirth; HBB, Helping Babies Breathe; IND, institutional newborn death; LDHF, low-dose high-frequency; MCQ, multiple-choice questionnaire; MEBCI, Making Every Baby Count Initiative; MNH, maternal and neonatal health; MIST, Multidisciplinary In-situ Simulation Training; NGO, non-governmental organisation; NICU, neonatal intensive care unit; NRP, Neonatal Resuscitation Program; NRT, Neonatal Resuscitation Training; OSCE, Objective Structured Clinical Examination; PPH, postpartum haemorrhage; PPV, positive pressure ventilation; QI, quality improvement; SSN, Safa Sunaulo Nepal; ToT, Training of Trainers.*

**References**

ABOU-ZAMZAM, A., MCCAW, J., NIARISON, H. R., RAVELOJAONA, V. A. & SHILKOFSKI, N. 2023. Cross-sectional study in Madagascar demonstrates efficacy of virtual mentoring and flipped classroom modifications of neonatal resuscitation programme Helping Babies Breathe. *Acta paediatrica (Oslo, Norway : 1992),* 112**,** 1783-1789.

BAMERI, F., GHADERI, R., ABOUBAKRI, O. & HEYDARIKHAYAT, N. 2024. Effect of continuous workshop training of the helping babies breathe program on the retention of midwives' knowledge and skills: A clinical trial study. *Nurse Educ Pract,* 78**,** 104020.

BAUMGARTNER, J. N., HEADLEY, J., KIRYA, J., GUENTHER, J., KAGGWA, J., KIM, M. K., ALDRIDGE, L., WEILAND, S. & EGGER, J. 2021. Impact evaluation of a maternal and neonatal health training intervention in private Ugandan facilities. *Health Policy & Planning,* 36**,** 1103-1115.

BRATHWAITE, K. P., BRYCE, F., MOYER, L. B., ENGMANN, C., TWUM-DANSO, N. A. Y., KAMATH-RAYNE, B. D., SROFENYOH, E. K., UCER, S., BOADU, R. O. & OWEN, M. D. 2020. Evaluation of two newborn resuscitation training strategies in regional hospitals in Ghana. *Resusc. Plus,* 1-2.

BRIGGS, D. C., ENEH, A. U. & ALIKOR, E. A. D. 2021. Basic neonatal resuscitation: retention of knowledge and skills of primary health care workers in Port Harcourt, Rivers State, Southern Nigeria. *Pan Afr Med J,* 38**,** 75.

CETINKAYA, S., TURKOGLU, B., DOGAN, E. & KARA, M. 2022. Examining the Knowledge Level of the Nurses and Midwives Had Neonatal Resuscitation Program (NRP) Practitioner Training Course. *J Multidiscip Healthc,* 15**,** 281-288.

CHALISE, M., DHUNGANA, R. P., VISICK, M. K. & CLARK, R. B. 2022. Assessing the effectiveness of newborn resuscitation training and skill retention program on neonatal outcomes in Madhesh Province, Nepal.

CHAN, N. H. M., MERALI, H. S., MISTRY, N., KEALEY, R., CAMPBELL, D. M., MORRIS, S. K. & DATA, S. 2023. Utilization of a novel mobile application, “HBB Prompt”, to reduce Helping Babies Breathe skills decay. *PLOS Global Public Health,* 3.

CHAULAGAIN, D. R., K, C. A., WRAMMERT, J., BRUNELL, O., BASNET, O. & MALQVIST, M. 2021a. Effect of a scaled-up quality improvement intervention on health workers' competence on neonatal resuscitation in simulated settings in public hospitals: A pre-post study in Nepal. *PLoS One,* 16**,** e0250762.

CHAULAGAIN, D. R., MALQVIST, M., BRUNELL, O., WRAMMERT, J., BASNET, O. & KC, A. 2021b. Performance of health workers on neonatal resuscitation care following scaled-up quality improvement interventions in public hospitals of Nepal - a prospective observational study. *BMC Health Serv Res,* 21**,** 362.

CHINBUAH, M. A., TAYLOR, M., SERPA, M., MAZIA, G., COFIE, P. K., KWARAH, W., DAWSON, S., NELSON, B. D. & ENGMANN, C. 2020. Scaling up Ghana's national newborn care initiative: integrating 'helping babies breathe' (HBB), 'essential care for every baby' (ECEB), and newborn 'infection prevention' (IP) trainings. *BMC Health Services Research,* 20**,** 739.

CLARK, R. B., CHALISE, M., VISICK, M. K., GHOSH, V. & DHUNGANA, R. 2023a. Scale-Up of a Newborn Resuscitation Capacity-Building and Skill Retention Program Associated With Improved Neonatal Outcomes in Gandaki Province, Nepal. *Glob Health Sci Pract,* 11.

CLARK, R. B., DHUNGANA, R., CHALISE, M. & VISICK, M. K. 2023b. Scale Up of Neonatal Resuscitation Training and Skill Retention in Five Provinces of Nepal. *Asia-Pacific Journal of Public Health,* 35**,** 381-387.

DATA, S., MIRETTE, D., CHEROP, M., BAJUNIRWE, F., KYAKWERA, C., ROBINSON, T., JOSEPHINE, N. N., ABESIGA, L., NAMATA, T., BRENNER, J. L., SINGHAL, N., TWINE, M., WISHART, I., MCINTOSH, H. & CHENG, A. 2022. Peer Learning and Mentorship for Neonatal Management Skills: A Cluster-Randomized Trial. *Pediatrics,* 150.

DHUNGANA, R., CHALISE, M., VISICK, M. K. & CLARK, R. B. 2024. A hybrid approach to skill retention following neonatal resuscitation training: Assessing effectiveness. *Journal of Neonatal-Perinatal Medicine,* 17**,** 555-564.

DING, X., WANG, L., MSELLEM, M. I., HU, Y., QIU, J., LIU, S., ZHANG, M., ZHU, L. & LATOUR, J. M. 2021. Evaluation of a Neonatal Resuscitation Training Programme for Healthcare Professionals in Zanzibar, Tanzania: A Pre-post Intervention Study. *Front Pediatr,* 9**,** 693583.

ERSDAL, H., MDOE, P., MDUMA, E., MOSHIRO, R., GUGA, G., KVALØY, J. T., BUNDALA, F., MARWA, B. & KAMALA, B. 2023. “Safer Births Bundle of Care” Implementation and Perinatal Impact at 30 Hospitals in Tanzania—Halfway Evaluation. *Children,* 10.

EZENWA, B. N., UMOREN, R., FAJOLU, I. B., HIPPE, D. S., BUCHER, S., PURKAYASTHA, S., OKWAKO, F., ESAMAI, F., FELTNER, J. B., OLAWUYI, O., MMBOGA, A., NAFULA, M. C., PATON, C. & EZEAKA, V. C. 2022. Using Mobile Virtual Reality Simulation to Prepare for In-Person Helping Babies Breathe Training: Secondary Analysis of a Randomized Controlled Trial (the eHBB/mHBS Trial). *JMIR Med Educ,* 8**,** e37297.

FARHADI, R., AZANDEHI, B. K., AMUEI, F., AHMADI, M., ZAZOLY, A. Z. & GHORBANI, A. A. 2023. Enhancing residents' neonatal resuscitation competency through team-based simulation training: an intervention educational study. *BMC medical education,* 23**,** 743.

FRITZ, J., MONTOYA, A., LAMADRID-FIGUEROA, H., FLORES-PIMENTEL, D., WALKER, D., TREVIÑO-SILLER, S., GONZÁLEZ-HERNÁNDEZ, D. & MAGAÑA-VALLADARES, L. 2020. Training in obstetric and neonatal emergencies in Mexico: effect on knowledge and self-efficacy by gender, age, shift, and profession. *BMC Med Educ,* 20**,** 97.

GAMTESSA, L. C., TIYARE, F. T. & KEBEDE, K. M. 2020. Evaluation of helping babies breathe and essential care for every baby training in southern nations nationalities and people’s region, Ethiopia: applying a Kirkpatrick training evaluation model. *BMC Research Notes,* 13.

HATAMLEH, R., ABUJILBAN, S., ABUHAMMAD, S., ARIAG, D. M. A. & JOSEPH, R. A. 2021. Effect of the Helping Babies Breathe Training Program: An Experimental Study on Jordanian Midwives' Knowledge and Skills. *Journal of Perinatal & Neonatal Nursing,* 35**,** E11-E17.

HEARD STITTUM, A. J., EDWARDS, E. M., ABAYNEH, M., GEBREMEDHIN, A. D., HORN, D., BERKELHAMER, S. K. & EHRET, D. E. Y. 2023. Impact of an Educational Clinical Video Combined with Standard Helping Babies Breathe Training on Acquisition and Retention of Knowledge and Skills among Ethiopian Midwives. *Children (Basel),* 10.

HIGGINS, B. V., MEDVEDEV, M. M., SPINDLER, H., GHOSH, R., LONGKUMER, O., COHEN, S. R., DAS, A., GORE, A., MAHAPATRA, T. & WALKER, D. M. 2020. Cohort study of neonatal resuscitation skill retention in frontline healthcare facilities in Bihar, India, after PRONTO simulation training. *BMJ Paediatrics Open,* 4.

INNERDAL, M., SIMAGA, I., DIALL, H., EIELSEN, M., NIERMEYER, S., EIELSEN, O. & SAUGSTAD, O. D. 2021. Reduction in perinatal mortality after implementation of HBB training at a district hospital in Mali. *J. Trop. Pediatr.,* 66**,** 315-321.

KANKINDI, F., MUHAYIMANA, A., MUJAWIMANA, P., DUSABE, R., NSENGIYUMVA, R. & MUKESHIMANA, M. 2020. Retention of Helping Babies Breathe Training among Midwives and Nurses at Four Rwandan Health Centers. *Rwanda Journal of Medicine and Health Sciences,* 3**,** 250-260.

LIMA, R. O., MARBA, S. T. M., ALMEIDA, M. F. B. & GUINSBURG, R. 2023. Impact of resuscitation training program on neonatal outcomes in a region of high socioeconomic vulnerability in Brazil: an interventional study. *J Pediatr (Rio J),* 99**,** 561-567.

MAYER, M. M., XHINTI, N., MASHAO, L., MLISANA, Z., BOBOTYANA, L., LOWMAN, C., PATTERSON, J., PERLMAN, J. M. & VELAPHI, S. 2022. Effect of Training Healthcare Providers in Helping Babies Breathe Program on Neonatal Mortality Rates. *Frontiers in Pediatrics,* 10.

MUBEEN, K., BAIG, M., ABBAS, S., ADNAN, F., LAKHANI, A., BHAMANI, S. S., REHMAN, B., SHAHID, S. & JAN, R. 2021. Helping babies breathe: assessing the effectiveness of simulation-based high-frequency recurring training in a community-based setting of Pakistan. *BMC Pediatrics,* 21**,** 555.

MUNIR, Z., HAYAT, S., FATIMA, B., ANWAR, M., QADIR, M. & WAHEED, I. 2021. Improvement in knowledge of healthcare professionals attending neonatal life support (NeoLiS) training workshop. *Journal of the Pakistan Medical Association,* 71**,** 1996-1999.

MUNYAW, Y., GIDABAYDA, J., YECONIA, A., GUGA, G., MDUMA, E. & MDOE, P. 2022. Beyond research: improved perinatal care through scale-up of a Moyo fetal heart rate monitor coupled with simulation training in northern Tanzania for helping babies breathe. *BMC Pediatrics,* 22**,** 1-9.

NEUPANE, D., SHARMA, A., THUKRAL, A., SANKAR, M. J., AGARWAL, R. & DEORARI, A. K. 2022. Simulation Based vs Conventional Training for Initial Steps in Delivery Room Care of Preterm Neonates: An Open Label Randomized Trial. *Indian Pediatr,* 59**,** 847-851.

NISHIMWE, A., IBISOMI, L., NYSSEN, M. & CONCO, D. N. 2021. The effect of an mLearning application on nurses’ and midwives’ knowledge and skills for the management of postpartum hemorrhage and neonatal resuscitation: pre–post intervention study. *Human Resources for Health,* 19.

NISHIMWE, A., IBISOMI, L., NYSSEN, M. & CONCO, D. N. 2022. The effect of a decision-support mHealth application on maternal and neonatal outcomes in two district hospitals in Rwanda: pre – post intervention study. *BMC Pregnancy and Childbirth,* 22.

NVONAKO, H., OJEE, E., MASIKA, M., SANDIE, A., WAMALWA, D. & WASUNNA, A. 2022. Effect of in-hospital training in newborn resuscitation on the competence of health-care workers in resuscitating newborn infants at birth at Mboppi Baptist Hospital, Douala, Cameroon. *Pan African Medical Journal,* 42.

NYIRINGANGO, G., KERR, M., BABENKO-MOULD, Y., KANAZAYIRE, C. & NGABONZIMA, A. 2021. Assessing the impact of mentorship on knowledge about and self-efficacy for neonatal resuscitation among nurses and midwives in Rwanda. *Nurse education in practice,* 52**,** 103030.

ODONGKARA, B., TYLLESKÄR, T., PEJOVIC, N., ACHORA, V., MUKUNYA, D., NDEEZI, G., TUMWINE, J. K. & NANKABIRWA, V. 2020. Adding video-debriefing to Helping-Babies-Breathe training enhanced retention of neonatal resuscitation knowledge and skills among health workers in Uganda: a cluster randomized trial. *Glob Health Action,* 13**,** 1743496.

PATTERSON, J. K., ISHOSO, D., EILEVSTJØNN, J., BAUSERMAN, M., HAUG, I., IYER, P., KAMATH-RAYNE, B. D., LOKANGAKA, A., LOWMAN, C., MAFUTA, E., MYKLEBUST, H., NOLEN, T., PATTERSON, J., TSHEFU, A., BOSE, C. & BERKELHAMER, S. 2023. Delayed and Interrupted Ventilation with Excess Suctioning after Helping Babies Breathe with Congolese Birth Attendants. *Children,* 10.

PATTERSON, J. K., ISHOSO, D., LOKANGAKA, A., IYER, P., LOWMAN, C., EILEVSTJØNN, J., HAUG, I., KAMATH-RAYNE, B. D., MAFUTA, E., MYKLEBUST, H., NOLEN, T., TSHEFU, A., BOSE, C. & BERKELHAMER, S. 2025. Neonatal outcomes and resuscitation practices following the addition of heart rate-guidance to basic resuscitation. *PLoS ONE,* 20.

SPIES, L. A., RILEY, C., NAIR, R., HUSSAIN, N. & REDDY, M. P. 2022. High-Frequency, Low-Dose Education to Improve Neonatal Outcomes in Low-Resource Settings: A Cluster Randomized Controlled Trial. *Advances in Neonatal Care (Lippincott Williams & Wilkins),* 22**,** 362-369.

STORDAL, K., EILEVSTJONN, J., MDUMA, E., HOLTE, K., THALLINGER, M., LINDE, J., MDOE, P., KIDANTO, H. & ERSDAL, H. L. 2020. Increased perinatal survival and improved ventilation skills over a five-year period: An observational study. *PloS one,* 15**,** e0240520.

TADESSE, M., HALLY, S., RENT, S., PLATT, P. L., EUSTERBROCK, T., GEZAHEGN, W., KIFLE, T., KUKORA, S. & POLLACK, L. D. 2021. Effect of a Low-Dose/High-Frequency Training in Introducing a Nurse-Led Neonatal Advanced Life Support Service in a Referral Hospital in Ethiopia. *Frontiers in Pediatrics,* 9.

TANEJA, G., SARIN, E., BAJPAYEE, D., CHAUDHURI, S., VERMA, G., PARASHAR, R., CHAUDHRY, N., MOHANTY, J. S., BISHT, N., GUPTA, A., TOMAR, S. S., PATEL, R., SRIDHAR, V. S., JOSHI, A., RATHI, C., BASWAL, D., GUPTA, S. & GERA, R. 2021. Care Around Birth Approach: A Training, Mentoring, and Quality Improvement Model to Optimize Intrapartum and Immediate Postpartum Quality of Care in India. *Global health, science and practice,* 9**,** 590-610.

TEH, K. H., LEE, I. L. & HARIFFADZILAH, C. H. 2024. Evaluation of the retention of knowledge, skills and competency of post-neonatal resuscitation training among house officers. *The Medical journal of Malaysia,* 79**,** 393-396.

TOSIF, S., JATOBATU, A., MAEPIOH, A., GRAY, A., SOBEL, H., MANNAVA, P. & DUKE, T. 2020. Healthcare worker knowledge and skills following coaching in WHO early essential newborn care program in the Solomon Islands: a prospective multi-site cohort study. *BMC Pregnancy and Childbirth,* 20**,** 84.

UMOREN, R., BUCHER, S., HIPPE, D. S., EZENWA, B. N., FAJOLU, I. B., OKWAKO, F. M., FELTNER, J., NAFULA, M., MUSALE, A., OLAWUYI, O. A., ADEBOBOYE, C. O., ASANGANSI, I., PATON, C., PURKAYASTHA, S., EZEAKA, C. V. & ESAMAI, F. 2021. eHBB: a randomised controlled trial of virtual reality or video for neonatal resuscitation refresher training in healthcare workers in resource-scarce settings. *BMJ Open,* 11**,** e048506.

UMUNYANA, J., SAYINZOGA, F., RICCA, J., FAVERO, R., MANARIYO, M., KAYINAMURA, A., TAYEBWA, E., KHADKA, N., MOLLA, Y. & KIM, Y. M. 2020. A practice improvement package at scale to improve management of birth asphyxia in Rwanda: a before-after mixed methods evaluation. *BMC Pregnancy Childbirth,* 20**,** 583.

VADLA, M. S., MDOE, P., MOSHIRO, R., HAUG, I. A., GOMO, Ø., KVALØY, J. T., OFTEDAL, B. & ERSDAL, H. 2022a. Neonatal Resuscitation Skill-Training Using a New Neonatal Simulator, Facilitated by Local Motivators: Two-Year Prospective Observational Study of 9000 Trainings. *Children,* 9**,** 134.

VADLA, M. S., MDUMA, E. R., KVALØY, J. T., MDOE, P., HHOKI, B. H., SARANGU, S., MICHAEL, P., OFTEDAL, B. & ERSDAL, H. 2024. Increase in Newborns Ventilated Within the First Minute of Life and Reduced Mortality After Clinical Data-Guided Simulation Training. *Simulation in healthcare : journal of the Society for Simulation in Healthcare,* 19**,** 271-280.

VADLA, M. S., MOSHIRO, R., MDOE, P., EILEVSTJØNN, J., KVALØY, J. T., HHOKI, B. H. & ERSDAL, H. 2022b. Newborn resuscitation simulation training and changes in clinical performance and perinatal outcomes: a clinical observational study of 10,481 births. *Advances in Simulation,* 7**,** 1-11.

WILSON, G. M., AME, A. M., KHATIB, M. M., KHALFAN, B. S., THOMPSON, J. & BLOOD-SIEGFRIED, J. 2020. Helping Babies Breathe (2nd edition) implementation on a shoestring budget in Zanzibar, Tanzania. *Maternal Health, Neonatology and Perinatology,* 6.

XU, C., CHEUNG, P. Y., XUE, Y., CHOW, C. B., XIE, Q., DONG, C. & ZHANG, Q. 2022. Improved Neonatal Out comes by Multidisciplinary Simulation - A Contemporary Practice in the Demonstration Area of China. *Hong Kong Journal of Paediatrics,* 27**,** 61.
