# Supplementary material for: Effectiveness and sustainability of neonatal resuscitation training programs in reducing neonatal mortality rates in low- and middle-income countries: a systematic review
Source: Glob Health Action. 2026 Nov 5;19(1):2757387. doi: 10.1080/16549716.2026.2757387 (PMC13757925; doi:10.1080/16549716.2026.2757387)
Supplement: Supplementary file 3 [file ZGHA_A_2757387_SM7806.docx]

**Supplementary file 3: Risk of Bias and Methodological Quality of included studies using Mixed Methods Appraisal Tool**

Methodological Quality of included studies using Mixed Methods Appraisal Tool (MMAT) (n=52)

| **Quantitative randomised controlled trials n=8**  *Quality appraisal was conducted on articles that met the two MMAT screening questions: ‘Are there clear research questions?’ and ‘Does the collected data allow for addressing the research questions?’* | | | | | | |
| --- | --- | --- | --- | --- | --- | --- |
| **Author (year)** | **2.1. Is randomisation appropriately performed?** | **2.2. Are the groups comparable at baseline?** | **2.3. Are there complete outcome data?** | **2.4. Are outcome assessors blinded to the intervention provided?** | **2.5. Did the participants adhere to the assigned intervention?** | **Comments**  **(Summary of evaluation quality based on MMAT results and sample size / Reviewer’s determination of High (H) versus Moderate (M) or Low (L) overall risk of bias** |
| Data et al. (2022) | Yes | Yes | Yes | Yes | Can’t tell | The study shows **low overall risk of bias (L).** Well-designed cluster RCT with appropriate randomisation at HSD level to prevent contamination, balanced baseline characteristics, and high retention. Centralised blinded assessment by calibrated raters strengthens internal validity. The study used validated HBB/ECEB assessment tools and standardised scoring. Peer practice adherence is incompletely documented. |
| Ersdal et al. (2023) | Yes | Yes | Yes | No | Yes | The study shows **moderate overall risk of bias (M)**. It was large, well-designed stepped-wedge cluster RC. It had excellent data completeness and appropriate randomisation with minor protocol deviation. However, no blinding (not feasible for implementation study). This is a halfway evaluation; final conclusions pending completion of 3-year study. |
| Ezenwa et al. (2022) | Yes | No | Yes | Can’t tell | Can’t tell | The study shows **moderate to high overall risk of bias (M-H)**. The study's strengths include complete outcome data for pre-course assessments. However, lack of clarity regarding assessor blinding and uncertainty about intervention adherence. While randomisation was appropriate, the significant baseline imbalance in knowledge scores raises concerns about randomisation effectiveness or measurement reliability. |
| Heard Stittum et al. (2023) | Yes | Yes | Yes | Can’t tell | Can’t tell | The study shows **moderate overall risk of bias (M)**. Strengths include appropriate cluster randomisation, balanced baseline characteristics, excellent retention (100% at 7 months), and use of standardised HBB assessment tools with calibrated raters. However, low adherence to interventions and potential contamination may compromise the effectiveness of the intervention. Uncertainty about rater blinding reduces confidence in results. |
| Neupane et al. (2022) | Yes | Yes | Yes | Yes | Yes | The study shows **low overall risk of bias (L**). Small but well-designed study with rigorous randomisation, complete follow-up (100%), and blinded outcome assessment. Equal time allocation and facilitator-participant ratios between groups controlled for potential confounding. The use of validated assessment tools and blinded raters strengthens credibility, though the single-centre design may affect generalizability. |
| Odongkara et al. (2020) | Yes | Yes | Yes | Yes | Yes | The study shows **low overall risk of bias (L).** It applied rigorous cluster randomisation methodology with appropriate allocation concealment, blinded outcome assessment, and high retention rates. Baseline differences in years of service and monthly resuscitations were appropriately adjusted for in multivariable analysis. The use of standardised HBB assessment tools and blinded assessors strengthens internal validity. |
| Spies et al. (2022) | Yes | Yes | No | No | Can’t tell | The study shows **moderate-to-high overall risk of bias (M-H**). It had only 1 cluster per treatment arm (generally inadequate for cluster RCT). Loss to follow-up for the primary outcome was high, and no blinding of assessors. Results cannot be generalised despite some statistically significant findings. |
| Umoren et al. (2021) | Yes | Yes | Yes | Yes | Can’t tell | The study shows **low-to-moderate overall risk of bias (L-M).** Well-designed multicentre RCT with rigorous randomisation, high retention rates and blinded outcome assessment. The study's strengths include a large sample size, a standardised HBB curriculum, and the use of validated assessment tools. However, uncertainty about actual intervention adherence (frequency of VR/video access, potential cross-contamination) and the use of multiple assessments as additional training interventions may reduce confidence in isolating the intervention effect. |

| **Quantitative non-randomised n=42**  *Quality appraisal was conducted on articles that met the two MMAT screening questions: ‘Are there clear research questions?’ and ‘Does the collected data allow for addressing the research questions?’* | | | | | | |
| --- | --- | --- | --- | --- | --- | --- |
| **Author (year)** | **3.1. Are the participants representative of the target population** | **3.2 Are measurements appropriate regarding both the outcome and intervention (or exposure)?** | **3.3. Are there complete outcome data?** | **3.4. Are the confounders accounted for in the design and analysis?** | **3.5. During the study period, is the intervention administered (or exposure occurred) as intended?** | **Comments**  **(Summary of evaluation quality based on MMAT results and sample size / Reviewer’s determination of High (H) versus Moderate (M) or Low (L) overall risk of bias** |
| Abou-Zamzam et al. (2023) | Yes | Yes | Yes | Can’t tell | Yes | Study shows **moderate overall risk of bias (M).** The study employed standardised, validated assessment tools with multiple training modalities compared. However, significant baseline differences between comparison groups not adjusted for. Cross-sectional design limits causal inference. Small sample sizes for some comparisons. |
| Bameri et al. (2024) | No | Yes | Yes | Yes | Yes | The study shows **low-to-moderate overall risk of bias (L-M**). Complete follow-up, appropriate statistical methods for repeated measures, validated tools, with clear intervention protocol study. However, this was single-center study with small sample size (n=61), limited external validity reduces generalizability. |
| Baumgartner et al. (2021) | Yes | Yes | Yes | No | Yes | The study shows **moderate overall risk of bias (M)**. The study had adequate sample size for primary outcomes and high follow up rates. However, lack of control group, inability to use adjusted models, variable training attendance, and staff turnover limit causal inference. |
| Brathwaite et al. (2020) | Yes | Yes | Yes | No | No | The study shows **high overall risk of bias (H).** The study had large sample, multiple measurement approaches, and real-time clinical observations at GARH. However, the study had non-comparable designs across sites, major confounding from concurrent QI initiatives at GARH, incomplete outcome data at some sites, and variable implementation fidelity. The study also cannot isolate the effects of NRP training from other interventions. The study design was not research-oriented but rather implementation-oriented with multiple uncontrolled variables. |
| Briggs et al. (2021) | Yes | Yes | Yes | No | Yes | The study shows **moderate overall risk of bias (M)**. The study employed representative sample with complete follow-up, and appropriate validated measurement tools. However, the study had limited confounder control, no blinding of assessors, small sample for refresher training analysis. |
| Cetinkaya et al. (2022) | Can’t tell | Yes | Yes | No | Can’t tell | Study shows **high overall risk of bias (H).** The study measurements were appropriate. The intervention (NRP training) occurred as documented. However, unclear representativeness of the target population due to convenience sampling, no control group, inability to establish causality regarding the relationship between training timing and knowledge levels and potential selection bias as participants self-selected to take the original NRP course. The study design cannot account for confounders. Without baseline data or comparison groups, it's difficult to attribute knowledge levels solely to the NRP training. |
| Chalise et al. (2022) | Yes | Yes | Yes | No | Yes | The study shows **moderate overall risk of bias (M).** It had large sample size with participant selection across multiple facility types. It employed appropriate and validated measurement tools and complete outcome data with quality verification. However, it had no control/comparison group. Without a control group, the study cannot establish causality. |
| Chan et al. (2023) | Can’t tell | Yes | Yes | Can’t tell | No | Study shows **moderate-to-high overall risk of bias (M-H)**. The study used appropriate measurements. However, the study had very high attrition (41-42%) lost-to-follow-up in both groups, leaving small final sample size severely limiting statistical power. No control group, no control for confounders and unclear whether intervention was administered as intended given very low app usage. |
| Chaulagain, K, et al. (2021) | Yes | Yes | No | Yes | Yes | Study **shows moderate-to-high overall risk of bias (M-H)**. The study recruited participants from different hospitals with varying sizes. The study employed standardised measurement tools. However, attrition rate was high with less than 50% included in analysis due to incomplete paired data, but good confounder adjustment with logistic regression adjusted for hospital size, position, age, gender, experience, previous training. No control groups. Time between basic and refresher training varied by hospital. |
| Chaulagain, Malqvist, et al. (2021) | Yes | Yes | No | Yes | Yes | Study shows **moderate overall risk of bias (M).** The study had a large sample size across multiple sites, standardised data collection with trained observers, adjustment for key confounders, and clear temporal comparison (before/after intervention). However, >30% participants not observed which cause significant selection bias risk. No control groups. Cannot isolate which specific QI components were most effective. |
| Chinbuah et al. (2020) | Yes | Yes | Yes | No | Yes | Study shows **low-to-moderate overall risk of bias (L-M).** The study had large sample size with good representativeness and appropriate stratified analyses. Strong intervention fidelity through systematic implementation. Comprehensive outcome measurement with minimal attrition. However, the study had no control groups, even though scale and consistency of results across regions strengthens findings. |
| Dhungana et al. (2024) | Yes | Yes | Yes | No | No | The study shows **moderate-to-high overall risk of bias (M-H)**. The study had appropriate measurement tools and complete outcome data. However, the study had small sample size, no control group, inability to adjust for confounders, and significant implementation changes due to COVID-19. Authors acknowledge study not powered to detect mortality differences. |
| Ding et al. (2021) | Yes | Yes | Yes | No | Yes | Study shows **moderate overall risk of bias (M).** The study employed self-designed validated knowledge tests and standardized skills checklists with complete follow-up of all participants. However, the sample size was small, no control group, pre-post design, practice opportunities between trainings unmeasured, potential language barriers. |
| Farhadi et al. (2023) | Yes | Yes | Yes | Can’t tell | Yes | Study shows **moderate overall risk of bias (M).** The study employed standardised validated measurement tools and all participants completed all assessment points with structured interventions. However, this study had small sample size with no control group. No multivariate adjustment for confounders with descriptive statistics only. |
| Fritz et al. (2020) | Yes | Yes | No | Yes | Yes | The study shows **low-to-moderate overall risk of bias (L-M**). Large multi-site sample, rigorous statistical methods, comprehensive confounder adjustment, with validated measurement tools study. However, the study had 14% attrition with differential dropout by gender/cadre, no long-term follow-up beyond immediate post-test. |
| Gamtessa et al. (2020) | Yes | Yes | Yes | No | Yes | Study shows **moderate overall risk of bias (M)**. The study had strong immediate post-training assessment and the study design was appropriate for its stated objectives. However, the study had no control group, inability to assess long-term retention beyond immediate post-training, small sample size from only two hospitals limits generalizability, and no assessment of confounders. |
| Hatamleh et al. (2021) | Yes | Yes | Yes | Yes | Yes | Study shows **low-to-moderate overall risk of bias (L-M).** The study employed control group with groups comparable at baseline across all demographics. The study used validated, culturally adapted instruments with rigorous statistical testing including assumption checks. However, the study was non-randomised but well-controlled statistically with simulation-based outcomes only. The sample size was relatively small although adequately powered for large effect sizes. Control group received "no training" but paper earlier states "all participants received some type of training in neonatal resuscitation techniques but not the HBB training program" unclear what baseline training was. |
| Higgins et al. (2020) | Yes | Yes | Yes | No | Yes | Study shows **moderate overall risk of bias (M).** The study had participants from various centres with strong simulation-based measurements and clear intervention delivery. However, the study had incomplete outcome data due to staff turnover, inability to distinguish individual vs. facility-level retention, and unmeasured confounding from varying previous training exposure. |
| Innerdal et al. (2021) | Yes | Yes | Yes | No | Yes | The study shows **moderate overall risk of bias (M).** This was study with large sample, stable data collection methods, and complete outcome data. However, this study had no control, single hospital, cannot isolate HBB effects from equipment provision, security situation limited external oversight, some baseline differences in maternal risk factors. |
| Kankindi et al. (2020) | Yes | Yes | Yes | No | Yes | The study shows **moderate overall risk of bias (M**). The study completed outcome data and employed validated measurement tools with appropriate statistical tests. However, the study had limited geographic representation with purposive sampling may introduce selection bias. No drop out reported with modest confounder adjustment. |
| Lima et al. (2023) | Yes | Yes | Can’t tell | No | Yes | The study shows **moderate overall risk of bias (M)**. Good intervention standardisation with multi-site intervention across large geographic area. High participation rate. Multiple assessment timepoints. However, the study had no complete data on all participants at follow-up (39%) of original sample), potential selection bias (in 12-month data), limited confounder adjustment, and no control groups |
| Mayer et al. (2022) | Yes | Yes | Yes | No | No | The study shows **moderate-to-high overall risk of bias (M-H).** The study had large sample size with complete outcome data. However, the study lacks a control group, no adjustment for confounders (particularly important clinical risk factors), an inability to isolate HBB effects from other interventions, and variable implementation across facilities/regions. |
| Mubeen et al. (2021) | Yes | Yes | No | No | Yes | Study shows **moderate overall risk of bias (M).** The study employed validated measurement tools and consistent intervention delivery. However, the study had 21% attrition rate with completers-only analysis, no control group, small final sample size, and inability to detect significant differences in clinical mortality outcomes. Self-reported clinical data from CMWs' MIS subject to bias. |
| Munir et al. (2021) | Yes | Yes | Can’t tell | No | Yes | Study shows **moderate-to-high overall risk of bias (M-H).** The study had large sample size across multiple workshops. However, no clear accounting of missing data or exclusions, no adjustment for confounders with limited information on tool validation and reliability. No control group, and no specific details on workshop duration, content coverage, or quality assurance procedures. |
| Munyaw et al. (2022) | Yes | Yes | Yes | No | Yes | The study shows **moderate overall risk of bias (M)**. The study had large sample, complete outcome data from validated sources, appropriate measurements, and good implementation fidelity. However, the study had no control group, so it cannot rule out temporal trends or concurrent interventions that may have affected the outcomes. |
| Nishimwe et al. (2021) | Yes | Can’t tell | Yes | No | Yes | Study shows **moderate-to-high overall risk of bias (M-H).** The study had completed baseline and follow up data. Appropriate statistical methods. However, the sample size for the study was small, limits generalisability, with inadequate skills measurement. Cannot separate SDA effects from other learning exposures. No assessment of actual clinical practice changes or patient outcomes. |
| Nishimwe et al. (2022) | Yes | Yes | Can’t tell | No | Yes | Study shows **moderate-to-high overall risk of bias (M-H**). The study employed innovative use of mHealth technology with clear definition of clinical outcomes. However, the study had no control group do cannot establish causality. No adjustment for confounding variables despite group differences. Baseline and endline groups had different characteristics. Small sample size for clinical outcomes. |
| Nvonako et al. (2022) | Yes | Yes | Yes | No | Yes | The study shows **low-to-moderate overall risk of bias (L-M)**. The study had complete enrolment of eligible participants and high completion rate. It employed appropriate measurement tools. However, single-center limits generalizability. No control group and limited adjustment for confounders. |
| Nyiringango et al. (2021) | Yes | Yes | Yes | No | Yes | The study shows **moderate overall risk of bias (M).** Good sample size and completion rate with validated measurement tools study. However, the study had limited adjustment for confounders in main analyses and no control group. Intervention fidelity not independently verified. External program (not researcher-controlled) |
| Patterson et al. (2023) | No | Yes | Can’t tell | No | Yes | The study shows **moderate-to-high overall risk of bias (M-H).** The study employed direct observation using validated Liveborn app with rigorous observer training and complete observational data. However, the study had no pre-intervention data with single-facility dominance. No adjustment for confounders and no control group for the study, |
| Patterson et al. (2025) | Yes | Yes | Can’t tell | Yes | Yes | Study shows **moderate overall risk of bias (M).** The study had large sample size across 3 facilities. The study employed validated standardised measurement tools with adjusted confounders. However, there was incomplete observational data as convenience sample. The study had no control group. Baseline differences between epochs. |
| R. B. Clark et al. (2023) | Yes | Yes | Yes | No | Can’t tell | The study shows **moderate-to-high overall risk of bias (M-H).**  This was study with large sample across multiple facilities, prospective cohort design, appropriate statistical methods, and complete outcome tracking. However, no control group, the study cannot establish causality. Implementation was uneven. No adjustment for confounding variables. Acknowledged data quality issues (misclassification some data) |
| Robert Brent Clark et al. (2023) | Yes | Can’t tell | Yes | No | No | The study shows **moderate-to-high overall risk of bias (M-H).** This was massive scale in multi-province implementation study. Long implementation period with diverse facility types and settings. However, the study not designed as research study, used routinely collected data without research goals, no control group, incomplete baseline data, and significant variation in implementation quality across provinces. Authors acknowledge cannot confirm causality. No adjustment for confounders. |
| Stordal et al. (2020) | Yes | Yes | Yes | Yes | Yes | The study shows **low overall risk of bias (L)**. Large population-based sample study with comprehensive data collection. Long follow-up period. It had appropriate adjustment for confounders, complete outcome data and clear intervention delivery. However, the study had no control group and potential selection bias from user fees (decrease in deliveries due to user fees). |
| Tadesse et al. (2021) | Yes | Yes | Yes | No | Yes | The study shows **moderate overall risk of bias (M).** This was study with large sample, using validated data collection. Comprehensive intervention with multiple time points for outcome. However, sample represents single hospital site, limiting generalizability. No control group, the study cannot establish causality. No adjustment for confounders. The study cannot isolate training effects from equipment provision. |
| Taneja et al. (2021) | Yes | Yes | Yes | No | Yes | Study shows **moderate overall risk of bias (M).** The study had very large scale (141 facilities), comprehensive assessment framework, and independent external evaluation. However, the study lack of control group, inability to isolate intervention effects from parallel government programs, and reliance on weighted sample for endline assessment. State-level variation suggests differential implementation quality. |
| Teh et al. (2024) | Yes | Yes | No | No | Yes | Study shows **moderate-to-high overall risk of bias (M-H).** The study employed clear measurements using validated tools. However, the attrition rate was high (almost 30% by 9 months) loss to follow up. No control group, confounding by practice opportunities not addressed, and small sample size limits generalizability. |
| Tosif et al. (2020) | Yes | Yes | No | No | Yes | The study shows **moderate overall risk of bias (M)**. This was multi-site study with good representation for the context. It employed validated measurement tools and complete post-intervention follow-up. However, the sample size was small, with minimal confounder adjustment. The baseline assessment was limiter with variable timing to evaluation. |
| Vadla et al. (2024) | Yes | Yes | Yes | No | Can’t tell | The study shows **moderate overall risk of bias (M).** This was large sample, comprehensive data collection, appropriate outcome measurement. However, the study had no control group, no multivariate adjustment for confounders in most analyses, intervention intensity declined in postintervention period, single facility limits generalisability |
| Vadla, Mdoe, et al. (2022) | Yes | Yes | Yes | No | Yes | The study shows **moderate overall risk of bias (M)**. The study employed good objective outcome measurement through automated simulator. Strong documentation of intervention with continuous automated recording, participants tracked over employment period, attrition due to job changes documented. However, the study had no control groups with no ability o isolate motivator effect from other factors. No adjustment for changing participant composition. |
| Vadla, Moshiro, et al. (2022) | Yes | Yes | Yes | Yes | Yes | The study shows **low overall risk of bias (L).** The data collected 24/7 by trained research assistants (eliminating selection bias), objective biomedical monitoring data, very large sample (n=10,481), complete outcome data (99%), adjusted analysis for confounders, clear documentation. However, the study lack of control group, but before-after design with two intervention phases allows assessment of incremental effect. |
| Xu et al. (2022) | Yes | Yes | Yes | Yes | Yes | The study shows **low-to-moderate overall risk of bias (L-M).** The study had very large sample size, complete outcome data, adjusted analysis for confounders, and consistent intervention delivery. However, the study lacks a randomised control group, but a before-and-after design is appropriate given the organisational intervention. Increased patient vulnerability over time appropriately identified and adjusted for in analysis. |

| **Mixed methods studies n=2**  *Quality appraisal was conducted on articles that met the two MMAT screening questions: ‘Are there clear research questions?’ and ‘Does the collected data allow for addressing the research questions?’* | | | | | | |
| --- | --- | --- | --- | --- | --- | --- |
| **Author (year)** | **5.1. Is there an adequate rationale for using a mixed methods design to address the research question?** | **5.2. Are the different components of the study effectively integrated to answer the research question?** | **5.3. Are the outputs of the integration of qualitative and quantitative components adequately interpreted?** | **5.4. Are divergences and inconsistencies between quantitative and qualitative results adequately addressed?** | **5.5. Do the different components of the study adhere to the quality criteria of each tradition of the methods involved?** | **Comments**  **(Summary of evaluation quality based on MMAT results and sample size / Reviewer’s determination of High (H) versus Moderate (M) or Low (L) overall risk of bias** |
| Umunyana et al. (2020) | Yes | Yes | Yes | Can’t tell | **Moderate** for quantitative component; **Moderate** for qualitative component | The study shows **moderate overall risk of bias (M).** It had exemplary mixed methods integration, very large scale (172 facilities). The qualitative data effectively explained quantitative outcomes and identified mentorship as critical component. The limitations for quantitative were no control group and for qualitative data were interviews by project team (potential bias), no data saturation reported, and endpoint-only interviews. |
| Wilson et al. (2020) | Yes | Yes | Yes | Yes | **Moderate** for quantitative component; **High** for qualitative component | The study shows **moderate overall risk of bias (M)** (limited by weakest component per MMAT guidance. It had good rationale for mixed methods and effective integration of quantitative (knowledge/skills) and qualitative (focus group) data. The limitation for quantitative component were high attrition at 6 months and no control group, while qualitative were only one focus group, minimal methodological detail, no data saturation. |
